# Adult-born dentate granule cells promote hippocampal population sparsity

**DOI:** 10.1038/s41593-022-01176-5

**Published:** 2022-10-10

**Authors:** Stephen B. McHugh, Vítor Lopes-dos-Santos, Giuseppe P. Gava, Katja Hartwich, Shu K. E. Tam, David M. Bannerman, David Dupret

**Affiliations:** 1 Medical Research Council Brain Network Dynamics Unit, Nuffield Department of Clinical Neurosciences, University of Oxford, Oxford, UK; 2 Department of Experimental Psychology, University of Oxford, Oxford, UK

**Keywords:** Neuronal physiology, Neurogenesis

## Abstract

The dentate gyrus (DG) gates neocortical information flow to the hippocampus. Intriguingly, the DG also produces adult-born dentate granule cells (abDGCs) throughout the lifespan, but their contribution to downstream firing dynamics remains unclear. Here, we show that abDGCs promote sparser hippocampal population spiking during mnemonic processing of novel stimuli. By combining triple-(DG-CA3-CA1) ensemble recordings and optogenetic interventions in behaving mice, we show that abDGCs constitute a subset of high-firing-rate neurons with enhanced activity responses to novelty and strong modulation by theta oscillations. Selectively activating abDGCs in their 4–7-week post-birth period increases sparsity of hippocampal population patterns, whereas suppressing abDGCs reduces this sparsity, increases principal cell firing rates and impairs novel object recognition with reduced dimensionality of the network firing structure, without affecting single-neuron spatial representations. We propose that adult-born granule cells transiently support sparser hippocampal population activity structure for higher-dimensional responses relevant to effective mnemonic information processing.

## Abstract (web summary)

McHugh et al. combine triple-(DG-CA3-CA1) ensemble recordings and optogenetic manipulations in the mouse hippocampus to show that adult-born granule cells transiently support sparser population activity for effective mnemonic information processing.

## Main

To process information in memory, the hippocampus uses sparse population activity whereby only a small proportion of neurons are simultaneously recruited^1^. The DG supports this process by gating sensory information to the hippocampus, decorrelating these inputs into nonoverlapping patterns^2,3^. Intriguingly, in the mammalian brain, the DG produces new excitatory neurons throughout adulthood^4^. These abDGCs integrate to the hippocampal circuitry within a few weeks and support various hippocampal-dependent behaviors^5–10^. However, the network-level contribution made by abDGCs to hippocampal firing dynamics is unclear. This knowledge gap reflects in part an absence of in vivo electrophysiological ensemble recordings that characterize the spiking activity of identified abDGCs and reveal their influence on the population-level structure of firing activity in the DG and downstream hippocampus proper *Cornu Ammonis* (CA) regions.

Information processing for memory-guided behavior involves the cooperative spiking of hippocampal excitatory principal cells (PCs), namely, DG granule cells and CA pyramidal cells^1^. Individual hippocampal PC activities can be tuned to the animal’s position and the surrounding cues so that each explored environment recruits a discrete combination of PCs mapping that space^11^. Accordingly, abDGCs could have a primary role in representing information by computing firing maps as other dentate granule cells do^12–16^. While the discovery of hippocampal maps provides an important mechanistic foundation for the role of the hippocampus in memory, such internal representations further involve precise spike time relationships among PCs, and with respect to the theta-band (5–12 Hz) oscillations that dominate the local field potentials (LFPs) of the network during active behavior^1,17^. This temporally structured, theta-paced spiking supports the computation of sparse firing patterns nested in the time frames of individual theta cycles where relatively few neurons out of the entire PC population transiently cooperate (co-fire)^1^. By reducing (decorrelating) the overlap between hippocampal firing patterns, population sparsity could support discriminative responses to mnemonic stimuli, increasing capacity and minimizing interference between memories. Accordingly, abDGCs may not solely represent information but also assist the organization of the temporally structured firing activity of the hippocampal PC population where only a small proportion of neurons are jointly recruited (co-active) at any given time.

To investigate the network-level contribution of abDGCs to hippocampal firing dynamics, we combined extracellular multichannel recordings and optogenetic interventions to identify and manipulate abDGCs while simultaneously monitoring LFPs and neuronal ensembles from the DG, CA3 and CA1 of behaving mice. Our findings first show that abDGCs constitute a small subset of high-rate granule cells with enhanced firing response to novelty and low spatial selectivity, but stronger spike coupling to the phase of theta oscillations compared with the other DG, CA3 and CA1 PCs. During a transient critical window of ~4–7 weeks, but not ~9–12 weeks, post-neuronal birth, we further observed that abDGC activation increases the sparsity of hippocampal population firing. In contrast, optogenetic suppression of abDGC spiking decreases population sparsity along with a hippocampus-wide disinhibition of DG, CA3 and CA1 PCs, increasing single-neuron firing rates but without altering individual place representations. Importantly, suppressing abDGC spiking during a continuous novel object recognition (cNOR) memory task prevented decorrelation between CA3–CA1 firing patterns and impaired successful response to novelty. We propose that during a critical period of their maturation, adult-born granule cells constitute a subnetwork of high-firing neurons promoting sparse hippocampal population activity for effective mnemonic processing of new information.

## Results

### Characterizing spiking activity of adult-born granule cells

**Fig. 1:**
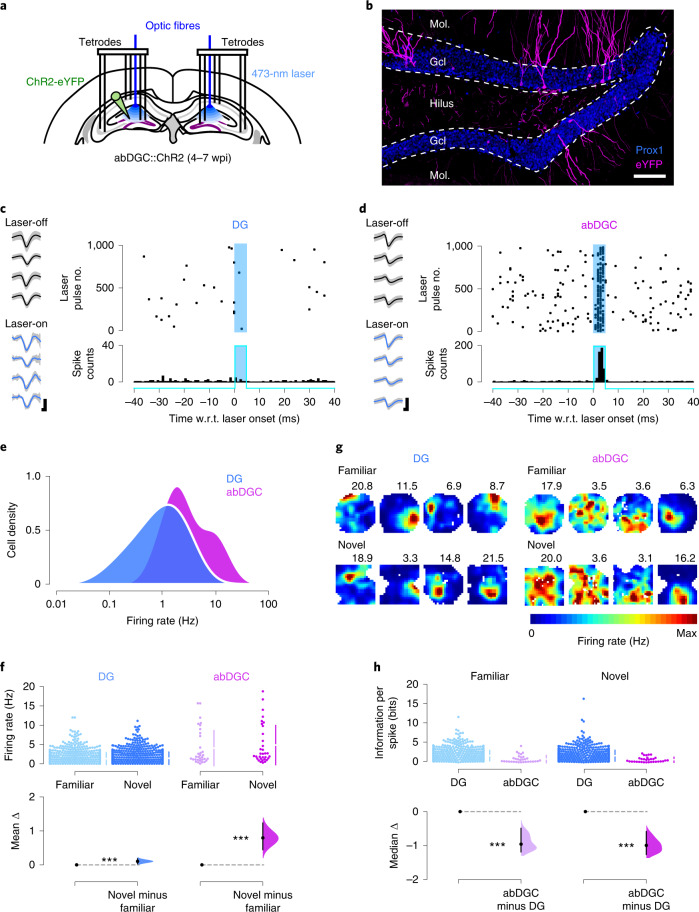
Adult-born hippocampal neurons constitute a subset of high-rate PCs. **a**, Triple-(DG-CA3-CA1) ensemble recording with DG light delivery. Adult-born DG cells transduced with ChR2-eYFP in adult (abDGC::ChR2) mice were identified using 5-ms blue-light pulses while monitoring DG, CA3 and CA1 spiking. **b**, ChR2-eYFP-expressing abDGCs at 7 wpi. DG granule cell nuclei stained with the transcription factor Prox1. Representative image from six mice. Scale bar, 100 µm. Gcl, granule-cell layer; Mol., molecular layer. **c**,**d**, An example non-opto-tagged DG PC (**c**) and an example opto-tagged abDGC (**d**): spikes (gray traces) with superimposed (black/blue traces) mean waveforms recorded across the four channels of their tetrode without (laser-off) and during (laser-on) light delivery (scale bar, 80 µV, 0.2 ms), raster-plot showing spike discharge with respect to (w.r.t.) light delivery (top right) and corresponding peri-stimulus time histogram (bottom right). **e**, Firing rate (log) distributions for non-opto-tagged DG PCs and abDGCs during laser-off sessions (kernel density estimates; see also Extended Data Fig. 1d). **f**, Cumming estimation plot showing the effect size for the differences in mean firing rates of non-opto-tagged DG PCs (*n* = 887) and abDGCs (*n* = 33) in eight mice during laser-off exploration of novel versus familiar environments. Upper panel: raw data points (each point represents one cell, with the gapped lines on the right as mean (gap) ± s.d. (vertical ends) for each environment). Lower panel: difference (Δ) in firing rates between novel versus familiar environment, computed from 5,000 bootstrapped resamples and with the difference-axis origin (dashed line) aligned to the mean firing rate in the familiar environment (black dot, mean; black ticks, 95% confidence interval; filled curve, sampling-error distribution). **g**, Spatial rate maps from four example non-opto-tagged DG PCs (left) and four example abDGCs (right) during laser-off exploration of familiar and novel environments. One cell per column; numbers indicate peak rate. **h**, Estimation plot (as **f**) showing spatial information in familiar and novel environments for abDGCs versus non-opto-tagged DG PCs. ***P < 0.001.

**Extended Data Fig. 1:**
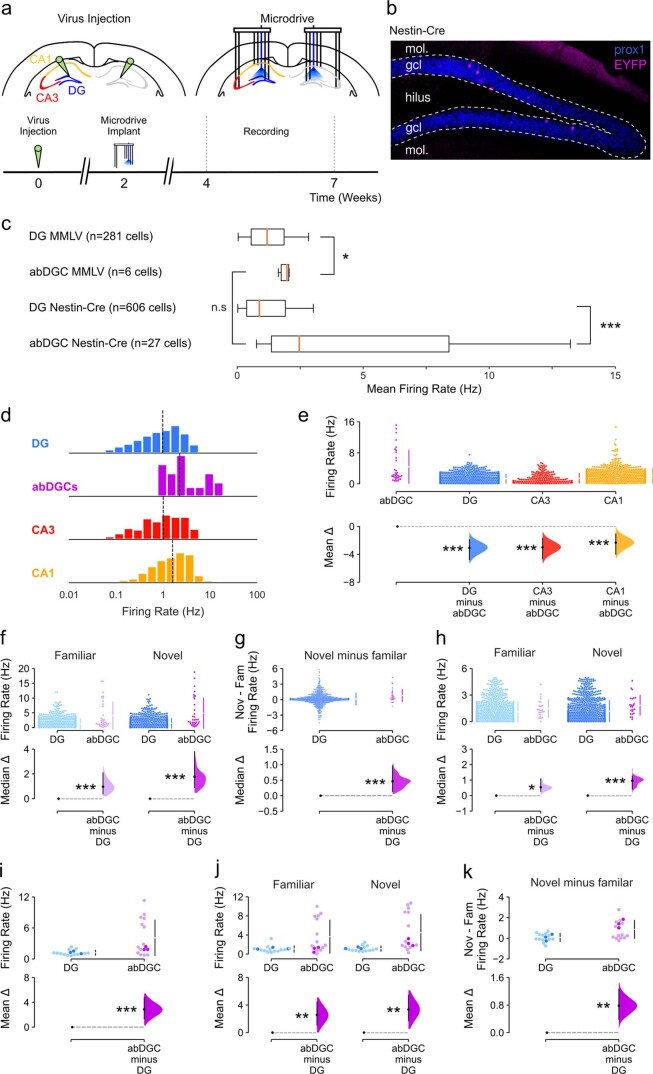
Experimental timeline and characteristics of abDGC firing. **(a)** Timeline of virus injection to deliver opsins into adult-born dentate granule cells (abDGCs), followed by microdrive implantation, containing 12 or 14 tetrodes (6 or 7 per hemisphere) to allow simultaneous recordings from DG, CA3 and CA1 neurons, and 2 optic fibres (1 per hemisphere) to allow light-delivery to the DG. Recordings took place 4-7 weeks after virus injection, meaning that abDGCs were in the so-called ‘critical period’ (4-7 weeks old)^89^ during the recording (including ‘opto-tagging’) sessions. **(b)** ChR2-eYFP-expressing abDGCs at 6-weeks post-injection (wpi) in a Nestin-cre mouse. Note: an example from a C57BL6/J mouse injected with a Moloney Murine Leukaemia retrovirus (MMLV) to deliver cre-recombinase into the offspring of dividing cells can be found in Fig. 1b. DG granule cell nuclei stained with the transcription factor Prox1. Representative image from six mice. Scale bar=100-µm. Granule-cell layer: gcl; molecular layer: mol. **(c)** The two methods of targeting abDGCs (Nestin-cre versus MMLV) produced comparable effects on firing rates: abDGCs had significantly higher firing rates compared to other DG principal cells (MMLV (2 mice): U = 414, p = 0.03; Nestin-cre (6 mice): U = 3106, p = 4.8e-08, two-sided Mann-Whitney tests), with no differences in abDGC firing rates between the two strategies (U = 102, p = 0.3, two-sided Mann-Whitney test). Box plots show the median (vertical orange line), box bounds are the lower and upper quartiles (Q1, Q3), and the lower / upper whiskers extend to Q1 - 0.75×IQR and Q3 + 0.75×IQR, respectively. For clarity, outliers are not shown but the total range across all DG PCs was 0.03 to 15.1 Hz. **(d,e)** Firing rates of abDGCs were significantly higher than the other DG, CA3 and CA1 principal cells. Panel d shows histograms of firing rates (0.2 Hz bin width) for these four principal cell populations, with the median firing rate shown by a dashed line. Panel **e** shows a Cumming estimation plot to visualize the effect size. Upper panel: distributions of raw data points (each point represents one cell, with the gapped lines on the right as mean (gap) ± s.d. (vertical ends) for each session). Lower panel: difference in firing rates between abDGCs versus other hippocampal PCs, computed from 5,000 bootstrapped re-samples and with the difference-axis (dashed line) origin aligned to the mean firing rate in the abDGCs (*black dot*, mean; *black ticks*, 95% confidence interval; *filled curve*: sampling error distribution; abDGC versus: DG: p = 0.001; CA3: p = 0.001; CA1: p = 0.001, two-sided permutation tests). Note that 0.01 Hz is the lowest firing rate for the principal cells we recorded in DG, CA3 and CA1 (median (IQR): 1.2 (0.56–2.38) Hz; versus abDGCs median (IQR): 2.3 (1.6–6.7) Hz; abDGC=33, DG = 887; CA3 = 201, CA1 = 748 cells in 8 mice). (**f**) Firing rates were higher in both familiar (p = 0.001) and novel (p = 0.001) environments in abDGCs (two-sided permutation tests). (**g**) For each neuron, we calculated a difference score based on the mean firing rate in each environment (novel minus familiar). Firing rates increased more from familiar to novel environments in abDGCs compared to other DG principal cells (p = 0.001, two-sided permutation test). (**h**) To ensure that the differences shown were not driven by a few high-rate abDGCs, we removed neurons with firing rates above 5-Hz. We found that even after this exclusion, abDGCs still had significantly higher firing rates than other DG principal cells in both the familiar (U = 7200, p = 0.014) and novel environments (U = 5717, p = 0.0008, two-sided Mann-Whitney tests). (**i-k**) In addition, we calculated aggregate statistics for each recording day that contained at least one opto-tagged abDGC (16 days out of 62; 13 from Nestin-cre mice, 3 from MMLV mice). We calculated the mean firing rate for all DG PCs or abDGCs recorded on that day so that each day generated paired data points. Using this approach, again we found significantly higher firing rates in abDGCs compared to other DG PCs. Panel i shows the mean rates estimated from whole recording days (p = 0.001, two-sided paired permutation test); panel j shows mean rates in the first familiar and novel sessions of each day (p = 0.006 for familiar, p = 0.002 for novel, two-sided paired permutation tests); panel k shows the difference from novel to familiar (p = 0.003, two-sided paired permutation test). In the upper panel of each plot, darker and lighter data points are from MMLV and Nestin-cre recording days, respectively. Panels f-k show Cumming estimation plots as described in panel **e**. ***p < 0.001, **p < 0.01, *p < 0.05.

To determine how abDGCs influence activity across the hippocampal network, we first characterized the spiking activity of abDGCs recorded in the mouse hippocampus during spatial exploration. To proceed, we transduced abDGCs with the blue- (473-nm) light-driven excitatory cation-channel Channelrhodopsin-2 (ChR2) (ChR2-eYFP) under the control of Cre-recombinase, using either a Moloney Murine Leukemia retrovirus or a transgenic (Nestin-Cre) mouse line strategy (Fig. 1a,b and Extended Data Fig. 1a,b). In these abDGC::ChR2 mice, subsequent implantation of tetrodes combined with optic fibers allowed parallel recordings of DG, CA3 and CA1 neurons with light delivery to the DG. We started each recording day by monitoring neuronal ensembles while mice explored familiar and novel open-field arenas without optogenetic intervention (‘laser-off’ sessions), before delivering brief (5-ms) laser pulses of blue light to optogenetically identify abDGCs amongst recorded neurons (‘laser-on’ sessions). We recorded 4–7 weeks after viral transduction (Extended Data Fig. 1a), an age range when abDGCs are anatomically integrated within the hippocampal circuitry^18–22^. From 920 PCs recorded in the DG of abDGC::ChR2 mice, we identified a total of 33 abDGCs using their optogenetically driven spiking response (Fig. 1c,d). This proportion (~3.6%) of opto-tagged abDGCs is consistent with studies reporting that abDGCs represent a small subpopulation of DG granule cells in the adult rodent brain^23,24^.

**Extended Data Fig. 2:**
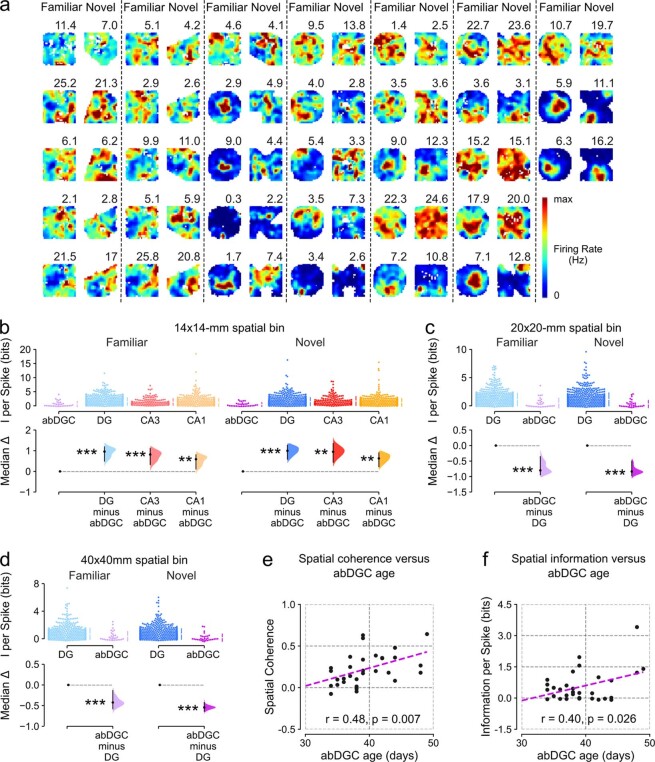
Spatial firing characteristics of abDGCs. **(a)** Spatial rate maps for the thirty-three abDGCs identified in abDGC::ChR2 mice and recorded during the familiar and novel environments. Peak firing rate (Hz) above each map. (**b**) Spatial information was lower in abDGCs compared to all other hippocampal PCs in both familiar and novel environments (familiar, abDGC versus: DG: p = 0.001; CA3: p = 0.001; CA1: p = 0.004, novel, abDGC versus: DG: p = 0.001; CA3: p = 0.003; CA1: p = 0.002, two-sided permutation tests, 14×14-mm spatial bin; abDGC=33, DG = 887; CA3 = 201, CA1 = 748 cells in 8 mice). Note that that all groups of hippocampal PCs contained significantly more spatial information than a null distribution, which was generated by shuffling spike times with respect to the location (p = 0.01 for abDGCs, p = 0.0001 for DG, CA3 and CA1, two-sided paired permutation tests for observed versus shuffled distributions, data not shown). (**c,d**) abDGCs still had lower spatial information than other DG principal cells when using different spatial bin sizes (20×20-mm, familiar: p = 0.001; novel: p = 0.0002; and 40×40-mm, familiar: p = 0.003; novel: p = 0.0002, two sided permutation tests). **(e,f)** The age of abDGCs was estimated from the number of days elapsed between virus injection and the date on which the abDGC was recorded (and opto-tagged). Both the spatial coherence (r = 0.48, p = 0.007) **(e)** and the spatial information (r = 0.40, p = 0.026) **(f)** carried by abDGCs were positively correlated with abDGC age (Pearson correlation, uncorrected for multiple comparisons). Panels b-d show Cumming estimation plots as described in Extended Data Fig. 1e. ***p < 0.001, **p < 0.01.

During laser-off exploration, the average firing rate of abDGCs was significantly higher than that of the other DG PCs (median (interquartile range): abDGCs, 2.3 (1.6–6.7) Hz; versus other DG PCs, 1.0 (0.4–1.9) Hz; *P* < 0.001, permutation test; Fig. 1e and Extended Data Fig. 1c–e). We observed this rate difference in both abDGC::ChR2 transduction strategies (Extended Data Fig. 1c). The firing rate of abDGCs was also higher when compared with CA3 and CA1 PCs (Extended Data Fig. 1d,e). Firing rates of both DG PCs and abDGCs increased in novel compared with familiar environments (*P* < 0.001, paired permutation tests; Fig. 1f and Extended Data Fig. 1f–k), but the novelty-enhanced firing of abDGCs was significantly greater (*P* = 0.001, permutation test; Extended Data Fig. 1g). By computing the firing maps of individual cells, we also observed that abDGC spiking carried significantly less spatial information than other DG PCs (Fig. 1h; *P* < 0.001, permutation test) and CA PCs (Extended Data Fig. 2a–d). Some abDGCs exhibited clear spatial tuning, which positively correlated with their age (Extended Data Fig. 2e,f). Thus, abDGCs constitute a subpopulation of high-rate DG PCs with an enhanced response to environmental novelty.

### abDGCs promote sparser hippocampal population activity

**Fig. 2:**
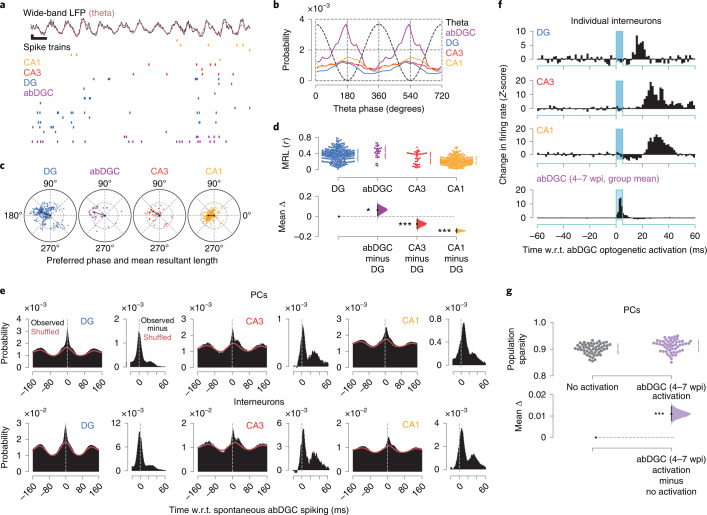
Adult-born hippocampal neurons are coupled to temporal network dynamics and increase population sparsity. **a**, Top: raw wide-band CA1 LFP trace (black) showing example population vectors of PC spikes nested in individual theta cycles (red, theta-filtered signal). Bottom: (color-coded) raster-plot of spike trains from CA1 (orange), CA3 (red) and DG (blue) PCs and abDGCs (purple) (one cell per row). Scale bars, 200 ms (horizontal), 200 µV (vertical). **b**, Mean spike probability of abDGCs and other DG, CA3 and CA1 PCs as a function of CA1 theta phase (dashed line, with two cycles for clarity). Spike probabilities normalized to the cell’s baseline spike rate. **c**, Polar plots showing theta preferred phase and modulation depth (mean resultant length, MRL) of individual abDGCs and other PCs in DG, CA3 and CA1 (dots), with group means (black lines). **d**, Corresponding estimation plot showing the effect size for differences in MRL (w.r.t. DG PCs). Upper and lower plots as in Fig. 1f. **e**, Spike discharge probability of DG, CA3 and CA1 PCs (top row) and interneurons (bottom row) w.r.t. spontaneous abDGC spike times. For each population: the left panel reports (laser-off) spiking probability referenced to spontaneous abDGC spikes (black histograms; with red line: the corresponding shuffled spike distribution with the original theta phase of each spike preserved); the right panel shows the observed minus the shuffled distribution. **f**, Change in instantaneous firing rate for three example interneurons in DG, CA3 and CA1 w.r.t. group mean optogenetic activation of 4–7-week-old abDGCs. **g**, Estimation plot showing sparsity of hippocampal PC population vectors before (gray) versus immediately following (purple) optogenetic activation of 4–7-week-old abDGCs. Each data point represents the mean sparsity for one recording session (*n* = 52 sessions in 8 mice). Upper and lower plots as in Fig. 1f. ***P < 0.001, *P < 0.05.

**Extended Data Fig. 3:**
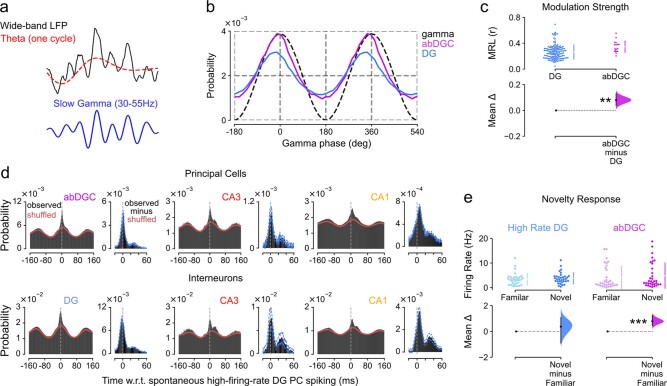
Additional analyses on abDGC temporal dynamics. **(a-c)** Spike coupling of abDGCs versus other DG PCs to slow gamma oscillations. **(a)** Top: Raw wide-band LFPs (black trace) recorded from the dentate granule layer showing an example single theta cycle (with red trace = theta filtered signal) nesting slow gamma (30-55 Hz) oscillations (with blue trace = corresponding slow gamma filtered signal from the black trace). **(b)** Mean spike probability of abDGCs (purple) versus other DG PCs (blue) as a function of ongoing slow gamma phase (dashed line, with two cycles for clarity). Both abDGCs and other DG PCs are locked to the peak of local slow gamma, but the strength of modulation was greater in abDGCs. Data taken from ‘laser-off’ periods. Spike probabilities were normalized to the cell’s baseline spike rate. **(c)** Corresponding estimation plot used to visualize the effect size for differences in the strength of modulation (mean resultant length, MRL) of abDGCs versus other DG PCs. The strength of slow gamma modulation was significantly greater for abDGCs compared to other DG PCs (p = 0.005, two-sided permutation test). **(d)** Spike discharge probability of DG, CA3 and CA1 PCs (top row) and interneurons (bottom row) w.r.t. spontaneous spike discharge of high-firing-rate DG PCs. High firing rate DG PCs were selected to best match the firing rates of concomitantly recorded abDGCs from each day. For each population: the left panel reports spiking probability referenced to spontaneous high-rate DC PC spikes (black histogram; with red line: the corresponding shuffled spike distribution with the original theta phase of each spike preserved); the right panel shows the observed minus the shuffled distribution. The dotted blue lines show the standard error of the mean. (Principal cells: abDGC n = 89, CA3 n = 84, CA1 n = 584; Interneurons: DG n = 232, CA3 n = 15, CA1 n = 104 cell-pairs in 8 mice). **(e)** Estimation plot showing the mean firing rate of the 33 abDGCs and their 33 rate-matched DG PCs in novel versus familiar environment. Only the abDGCs showed a significant increase in firing rate in the novel environment (p = 0.001, two-sided paired permutation test). Panels c,e show Cumming estimation plots as described in Extended Data Fig. 1e. ***p < 0.001, **p < 0.01.

We next investigated whether abDGC spiking is coupled to the temporal dynamics of hippocampal network activity and evaluated their contribution to population-level patterns. To do so, we first assessed spike time relationships to network oscillations. Notably, theta oscillations detected in the LFPs coordinate neuronal spiking across the network where theta-nested population vectors of PC spikes yield sparse firing structure during exploration (Fig. 2a)^1,17^. We examined the spike theta phase relationship of DG, CA3 and CA1 PCs with respect to the theta oscillations recorded from the CA1 pyramidal layer, calculating both the theta phase distribution of the spikes discharged by each population (Fig. 2b) and the theta phase preference of single neurons (Fig. 2c). The firing probability of abDGCs coincided with that of the other DG PCs, being at a maximum towards the end of the descending phase of the CA1 pyramidal layer theta reference (mean phase preference in laser-off periods: abDGCs, 162 ± 47°; other DGs, 157 ± 52°; *P* = 0.7, Watson–Wheeler test; with theta peak as zero-degree reference). Both subsets of DG PCs shared a theta phase space that overlapped with that of CA3 PCs, preceding the increased CA1 PC firing that marks the trough of theta waves. However, abDGCs showed significantly stronger theta phase modulation compared with the other PC populations (Fig. 2d; laser-off periods: *P* = 0.04 compared with other DG; *P* < 0.001 versus CA3 and CA1; permutation tests). Similarly, abDGCs exhibited stronger coupling to local slow gamma oscillations (Extended Data Fig. 3a–c). By showing that abDGCs discharge high-rate spikes that are temporally structured with respect to network oscillations, these findings suggest that the abDGC subpopulation could exert strong influence on downstream hippocampal activity.

To examine more directly the spike timing relationships between abDGCs and the other hippocampal populations, we computed group cross-correlations to assess the discharge probability of both PCs and fast-spiking interneurons in the DG, CA3 and CA1, with respect to abDGC spikes. In line with our theta LFP-unit analyses (Fig. 2b–d), the group cross-correlograms of individual abDGCs with DG, CA3 and CA1 PCs and interneurons showed strong theta modulation during laser-off periods (Fig. 2e). By computing temporally shifted spike controls to account for theta phase modulation, we further observed that both PCs and interneurons in DG, CA3 and CA1 exhibited a transient increase in firing with a time lag of 20–40 ms following abDGC spontaneously observed spikes (Fig. 2e; cross-correlograms from observed spike trains minus control cross-correlograms from theta phase shifted spike trains, using laser-off periods). A similar transient firing increase marked the theta cross-correlograms computed with a subset of DG cells obtained by matching their individual firing rates to those of simultaneously recorded abDGCs (Extended Data Fig. 3d); however, these other high-rate DG cells showed weaker firing response to novelty compared with their rate-matched abDGCs (Extended Data Fig. 3e), suggesting that under natural conditions, the abDGCs are more prone to influence downstream targets due to their higher response to novelty. We also found that optogenetic activation of 4–7-week-old abDGCs artificially entrained spiking activity of some DG, CA3 and CA1 interneurons (Fig. 2f; laser-on sessions), with a similar (~20–40-ms) temporal lag to that seen following spontaneous abDGC spiking (Fig. 2e; laser-off periods).

These results suggest that abDGCs could shape spike dynamics across the network, assisting the organization of temporally structured population patterns of downstream PCs by controlling their collective spiking activity via inhibitory interneurons. Such a tight excitation–inhibition balance could then promote the sparse structure of population firing thought to underlie many neural computations^25^. In line with this, we found that during open-field exploration, optogenetic activation of 4–7-week-old abDGCs (using 5-ms blue-light delivery to the DG) increased population sparsity of hippocampal PC firing patterns (Fig. 2g; *P* < 0.001, paired permutation test).

**Fig. 3:**
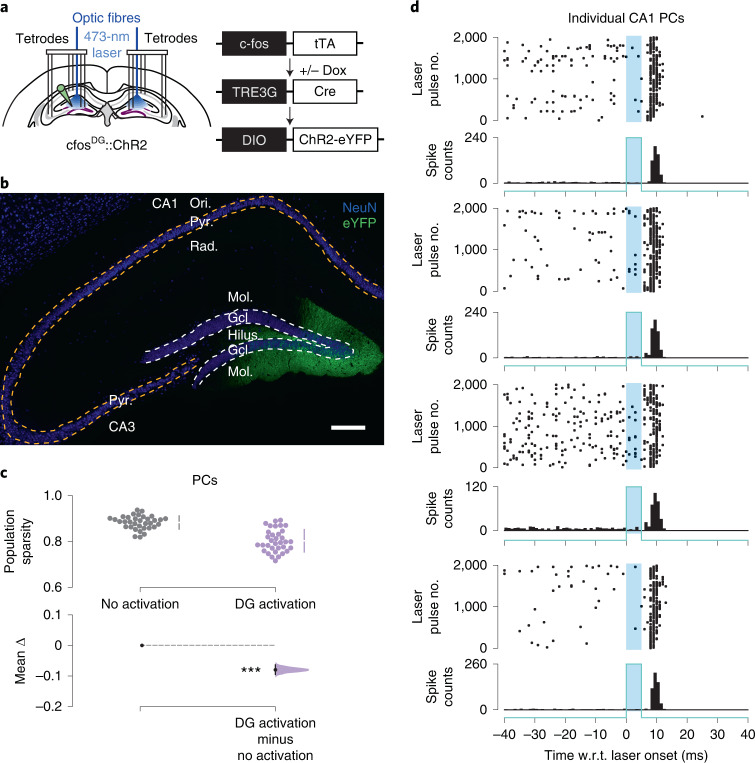
Activating a set of DG cells selected irrespective of their birthdate does not reproduce the abDGC sparsification function. **a**, CA ensemble recording with 5-ms blue-light delivery in c-fos^DG^::ChR2 mice to activate a subset of DG cells in a birthdate-independent manner. This ChR2-tagging approach leverages the activity-dependent expression of the tTA through the promoter of the c-fos immediate early gene by injecting the DG of c-fos–tTA transgenic mice with a TRE3G-Cre virus combined with the Cre-dependent ChR2-eYFP virus. **b**, ChR2-eYFP-tagged DG cells. Cell nuclei stained with the neuronal nuclear marker (NeuN). Representative image from four mice. Scale bar, 200 µm. The number of ChR2-eYFP-tagged cells in the dorsal DG of c-fos^DG^::ChR2 mice was sparse, with a trend for higher number of opsin-targeted cells compared with ChR2-expressing abDGCs in abDGC::ChR2 mice (abDGC::ChR2, 1,206.25 ± 61.35; c-fos^DG^::ChR2, 1,402.50 ± 73.07; Mann–Whitney *U* = 14.0; *P* = 0.1; *n* = 4 mice per group; mean ± s.e.m.). **c**, Estimation plot showing sparsity of CA population vectors before (gray) versus immediately following (purple) optogenetic activation. Each data point represents the mean sparsity for one recording session (*n* = 34 sessions in 3 mice). Upper and lower plots as in Fig. 1f. **d**, Spiking activity of four example simultaneously recorded CA1 PCs w.r.t. optogenetic activation. ***P < 0.001. Ori., Oriens; Pyr., Pyramidale; Rad., Radiatum.

DG cells can potently influence downstream network activity through ‘detonator synapses’ at mossy fiber terminals, discharging both excitatory and inhibitory CA3 neurons^26^. We thus tested whether the sparsification of CA population firing caused by abDGC activation could be replicated by activating another subpopulation of DG cells, when ChR2-expressing DG cells are not selected based on their birthdate. We used adult c-fos–tTA transgenic mice to leverage the activity-dependent expression of the tetracycline transactivator (tTA) through the promoter of the c-fos immediate early gene, thereby tagging a subset of DG cells with ChR2-eYFP in a birthdate-independent manner (Fig. 3a). To achieve this, we generated a viral construct carrying a tTA-dependent tetracycline-responsive element expressing the Cre-recombinase (TRE3G-Cre) combined with the same Cre-dependent ChR2-eYFP construct used in abDGC::ChR2 mice (Figs. 1 and 2). This dual-adeno-associated virus (AAV) targeting yielded c-fos^DG^::ChR2 mice where the transient removal of doxycycline (Dox) from the mouse diet allows the tTA to interact with the TRE3G element for DG cell tagging during spatial exploration of an open-field arena (Fig. 3a,b). In these c-fos^DG^::ChR2 mice, we then recorded neuronal ensembles during subsequent exploration of various open-field arenas, combined with 5-ms blue-light delivery to the DG to evaluate the consequences on the sparsity of population activity, as before. We found that this intervention altered CA population activity, but by significantly reducing sparsity (Fig. 3c; *P* < 0.001, paired permutation test) with feed-forward entrainment of CA spiking (Fig. 3d). Thus, while this result confirmed that activating a subpopulation of DG cells independently of their birthdate modulates downstream CA spiking, this was in the opposite direction to the network effect caused by optogenetic activation of 4–7-week-old abDGCs (Fig. 2g).

**Fig. 4:**
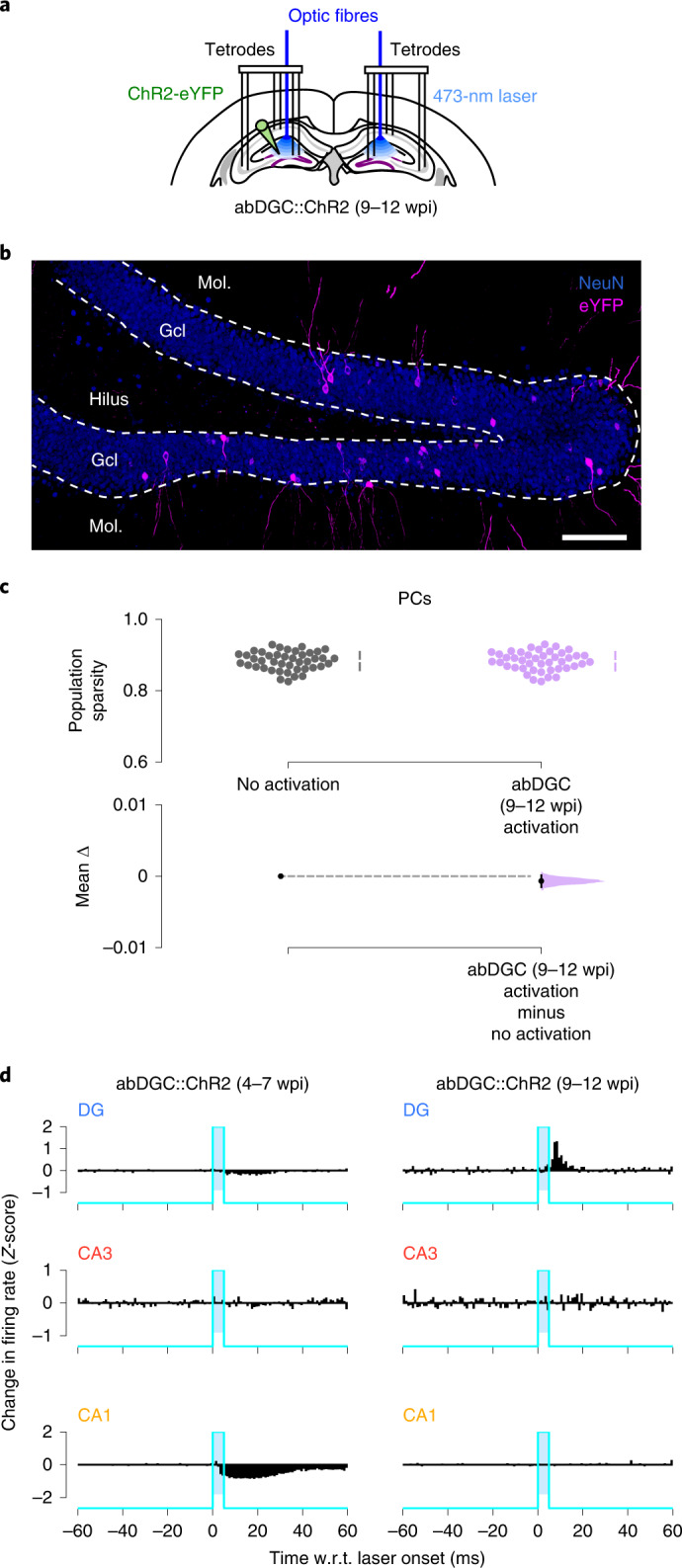
The sparsification function of abDGCs is restricted to a transient period of their maturation. **a**, Triple-(DG-CA3-CA1) ensemble recording with 5-ms blue-light delivery in abDGC::ChR2 mice at 9–12 wpi. **b**, ChR2-eYFP-expressing abDGCs at 9.5 wpi. DG granule cell nuclei stained with NeuN. Representative image from four mice. Scale bar, 100 µm. **c**, Estimation plot showing sparsity of PC population vectors before (gray) versus immediately following (purple) optogenetic activation of 9–12-week-old abDGCs. Each data point represents the mean sparsity for one recording session (*n* = 43 sessions in 3 mice). Upper and lower plots as in Fig. 1f. **d**, Group mean change in *Z*-scored instantaneous firing rate for the PC populations recorded in DG, CA3 and CA1 of abDGC::ChR2 mice, w.r.t. abDGC activation during 4–7 versus 9–12 wpi.

During the first weeks following their birth, abDGCs undergo important rearrangement in their pre- and postsynaptic connectivity, intrinsic properties and synaptic plasticity^18,27^, all events that could modulate the impact of abDGCs on hippocampal dynamics. To determine whether the observed effect on network sparsity depends upon abDGC activity during a transient (4–7-week) period, we next assessed whether activating mature abDGCs also alters hippocampal population sparsity. We conducted further DG-CA3-CA1 recordings in abDGC::ChR2 mice during the period of 9–12 weeks post-injection (wpi) (Fig. 4a,b). However, activating these older abDGCs with 5-ms blue-light pulses did not affect population sparsity during spatial exploration (Fig. 4c; *P* = 0.13, paired permutation test), in marked contrast to the increased sparsity found when activating abDGCs in their 4–7-week maturation period (Fig. 2g). Notably, while we found that activating 4–7-week-old abDGCs suppressed CA1 PC spiking, activating 9–12-week-old abDGCs was without effect at a population level (Fig. 4d). However, activating 9–12-week-old abDGCs did entrain other DG PCs (Fig. 4d), a local effect not seen during the 4–7-wpi period. These results demonstrate that the network effect of abDGCs evolves over time, with their ability to promote population sparsity restricted to a critical period of their maturation.

**Fig. 5:**
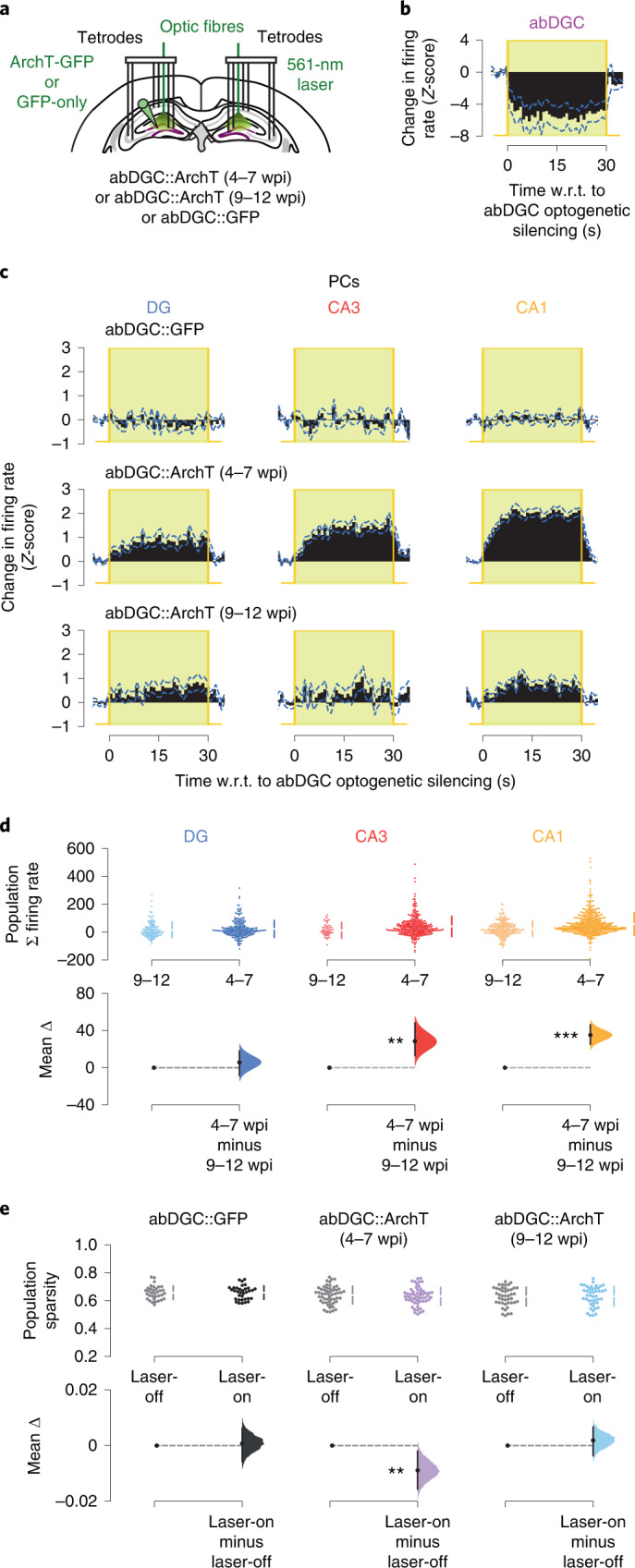
Silencing 4–7-week-old abDGCs increases PC spiking across the network and reduces population sparsity. **a**, Triple-(DG-CA3-CA1) ensemble recording with yellow (561-nm) DG light delivery. Adult-born DG cells transduced with GFP-only (abDGC::GFP) or ArchT-GFP (abDGC::ArchT), monitoring DG, CA3 and CA1 PCs in the following 4–7 or 9–12 weeks. **b**, Optogenetic silencing of abDGCs (*n* = 13 cells in 4 mice) in abDGC::ArchT mice (change in population mean firing rates (*Z*-scored); dashed blue line, s.e.m.). **c**, Change in population mean firing rates (*Z*-scored) for DG, CA3 and CA1 PCs during 4–7-week-old or 9–12-week-old abDGC silencing in abDGC::ArchT mice or equivalent laser-on periods in abDGC::GFP mice. **d**, Estimation plot showing the effect size for changes in firing rate of DG, CA3 and CA1 PCs during 4–7-week-old or 9–12-week-old abDGC silencing in abDGC::ArchT mice. Upper plot: each data point represents the *Z*-scored summed firing change for a PC. Lower plot: mean difference in summed firing rate between silencing abDGCs at 4–7 versus 9–12 wpi (abDGC::ArchT (4–7 wpi): DG *n* = 219, CA3 *n* = 290, CA1 *n* = 437 in 4 mice; abDGC::ArchT (9–12 wpi): DG *n* = 131, CA3 *n* = 56, CA1 *n* = 213 in 2 mice). **e**, Estimation plot showing sparsity of PC population vectors before (laser-off) versus following (laser-on) light onset in abDGC::GFP and abDGC::ArchT mice, for 4–7-week-old or 9–12-week-old abDGC silencing. Upper plot: each data point represents the mean sparsity for one recording session in either the laser-off or laser-on condition (abDGC::GFP: *n* = 34 sessions in 2 mice; abDGC::ArchT (4–7 wpi): *n* = 54 sessions in 4 mice; abDGC::ArchT (9–12 wpi): *n* = 44 sessions in 2 mice). Lower plot: mean difference in population sparsity between laser-off and laser-on epochs. Panels **d** and **e** show Cumming estimation plots as in Fig. 1f. ***P < 0.001, **P < 0.01.

**Extended Data Fig. 4:**
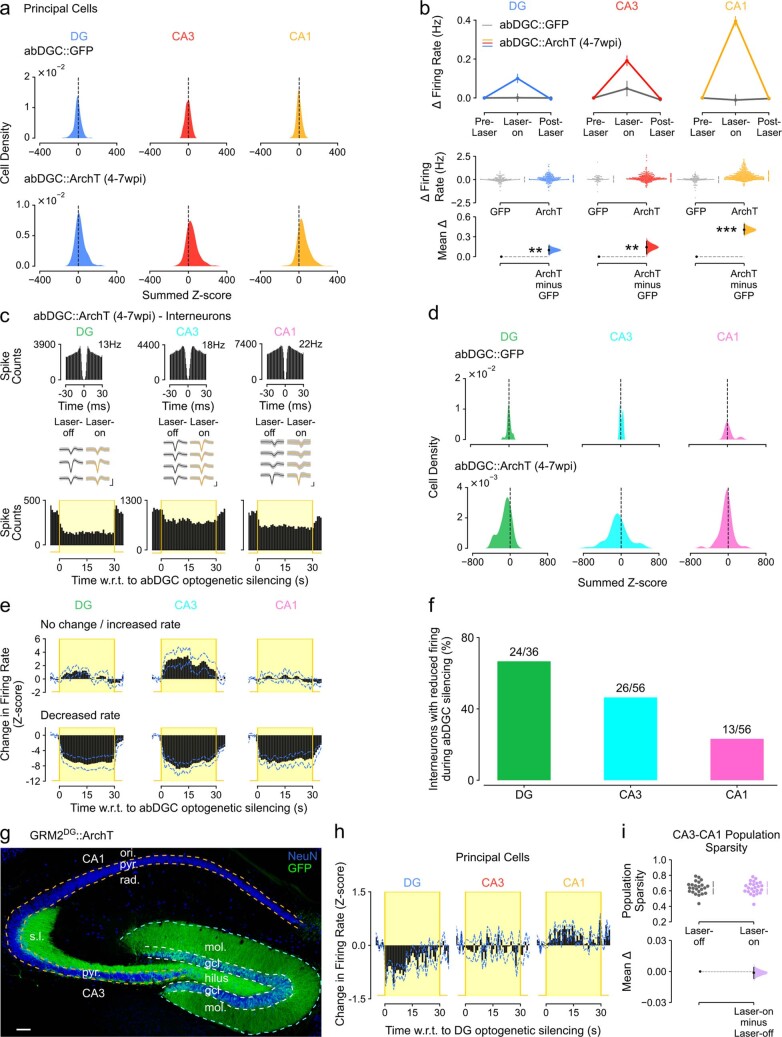
Silencing abDGCs alters firing dynamics of PCs and interneurons across the hippocampal network. **(a,b)** Silencing 4-7-week-old abDGCs increased firing rates in DG, CA3 and CA1 PCs. **(a)** Distributions of changes in hippocampal PC firing rates (summed z-scores) during laser-on periods, using kernel density estimate (k.d.e)^80^. In abDGC::GFP mice (top row), these distributions were symmetrical and tightly clustered around zero. However, in abDGC::ArchT mice (bottom row) these distributions were shifted to the right of zero, indicating that during abDGC silencing, the population firing rate of the other hippocampal PCs increased. **(b)** Top row: The mean absolute change in firing rates in PCs during laser-on periods compared to laser-off periods before (Pre-Laser) and after (Post-Laser) DG light delivery in both abDGC::GFP and abDGC::ArchT mice. Bottom row: Cumming estimation plot to visualize the effect size for differences in firing rate during the laser-on periods for abDGC::ArchT versus abDGC::GFP. Firing rates were significantly higher in abDGC::ArchT mice for all three subregions (DG: p = 0.004; CA3: p = 0.01; CA1: p = 0.001, two-sided permutation tests; abDGC::GFP: DG n = 147, CA3 n = 70, CA1 n = 124 cells in 2 mice; abDGC::ArchT (4-7wpi): DG n = 219, CA3 n = 290, CA1 n = 437 cells in 4 mice). **(c-f)** Silencing 4-7-week-old abDGCs caused reduced firing in some putative GABAergic interneurons. **(c)** Three example interneurons (one per column) in DG, CA3 and CA1 showing reduced firing during abDGC silencing: auto-correlograms (top left, with each cell’s mean firing rate on the right of each plot), spike waveforms during laser-off and laser-on sessions (top right), light-triggered cross-correlograms (bottom row). **(d)** Distributions of changes in hippocampal interneuron firing rates (k.d.e. on the summed z-scores) during laser-on periods. In abDGC::GFP mice (top row), these distributions were symmetrical and clustered around zero. However, in abDGC::ArchT mice (bottom row) these distributions were shifted to the left of zero, indicating that the overall interneuron population decreased its average firing rate during abDGC silencing. Yet, we noted some heterogeneity across firing responses of individual interneurons during abDGC silencing. **(e)** To visualize the heterogeneity seen across firing responses of single interneurons during abDGC silencing **(d)**, we isolated interneurons with significantly decreased rate (z-score below -2 on 40% of bins during laser-on periods; bottom row) versus the rest of the interneurons (top row). **(f)** The percentage of interneurons that significantly decreased their firing rate during laser-on periods in abDGC::ArchT mice, as in **(e)**, were: DG (66.7%), CA3 (46.4%) and CA1 (23.2%). **(g-i)** Silencing a larger fraction of DG PCs in Grm2^DG^::ArchT mice increased CA1 firing but did not affect CA3-CA1 sparsity. **(g)** ArchT-GFP-expressing dentate granule cells in a Grm2^DG^::ArchT mouse. Note the dense staining of DG dendrites in the molecular layer and DG axons in the hilus and stratum lucidum. Neuronal nuclei stained with NeuN. Representative image from three mice. Scale bar=100-µm. Granule-cell layer: gcl; molecular layer: mol; pyramidal layer: pyr; stratum oriens: ori; stratum radiatum: rad; stratum lucidum: s.l. **(h)** Change in population mean firing rates (z-scored) for DG, CA3, and CA1 PCs during DG silencing in Grm2^DG^::ArchT mice. There was no significant change in CA3 PC population firing but the decrease in DG population firing was significantly below zero, and the increase in CA1 population firing was significantly above zero (DG: t(231) = -3.5, p = 0.001; CA3: t(71) = -0.4, p = 0.7; CA1: t(244) = 2.3, p = 0.02, two-sided single-sample t-tests; DG n = 232, CA3 n = 70, CA1 n = 124 cells in 2 mice). **(i)** Estimation plot showing sparsity of CA PC population vectors before (Laser-off) versus immediately after DG silencing (Laser-on) in Grm2^DG^::ArchT mice (p = 0.7, two-sided paired permutation test). Each data point represents the mean CA sparsity for one recording session (n = 24 sessions in 3 mice). Panels b,i show Cumming estimation plots as described in Extended Data Fig. 1e. ***p < 0.001, **p < 0.01.

To further probe abDGCs’ network contribution to sparse population firing structure, we transduced abDGCs with either the yellow- (561-nm) light-driven optogenetic silencer Archaerhodopsin-T (ArchT) or the GFP-only control construct in separate groups of mice (Fig. 5a; abDGC::ArchT versus abDGC::GFP mice, respectively), subsequently monitoring DG, CA3 and CA1 ensembles with DG light delivery. Strikingly, silencing abDGCs during the 4–7-wpi period (Fig. 5b) increased the firing rates of DG, CA3 and CA1 PCs over the physiological range in abDGC::ArchT mice (Fig. 5c,d and Extended Data Fig. 4a,b; *P* < 0.0001, permutation tests for abDGC::ArchT (4–7-week-old) versus abDGC::GFP mice). This optogenetically enhanced firing of PCs occurred with the reduced firing of individual fast-spiking interneurons (Extended Data Fig. 4c–f), consistent with our findings above (Figs. 2 and 4). The effect of abDGC silencing on CA PC firing rates was substantially weaker during the 9–12-wpi versus the 4–7-wpi period (Fig. 5c,d; *P* < 0.006, permutation tests). In line with our observation that activating 4–7-week-old abDGCs promotes sparser hippocampal population patterns in abDGC::ChR2 mice (Fig. 2g versus Fig. 4c), abDGC silencing reduced PC population sparsity in abDGC::ArchT mice when performed during the 4–7-wpi period (Fig. 5e; *P* = 0.01, paired permutation test) but not in the 9–12-wpi period (Fig. 5e; *P* = 0.5, paired permutation test). DG yellow-light delivery in control abDGC::GFP mice did not alter hippocampal population sparsity (Fig. 5e; *P* = 0.8, paired permutation test). Moreover, silencing a much larger fraction of DG PCs in Grm2^DG^::ArchT mice (where DG granule cells of metabotropic-glutamate-receptor 2-Cre mice are targeted with ArchT) also did not affect CA population sparsity (Extended Data Fig. 4g–i), which further supports the selective influence of 4–7-week-old abDGCs on population sparsity. Collectively, these results show that abDGCs affect spiking activity across the hippocampal network and allowed us to hypothesize that abDGC-mediated population sparsity is important for hippocampal mnemonic function.

### abDGC-modulated hippocampal firing sparsity supports memory

**Extended Data Fig. 5:**
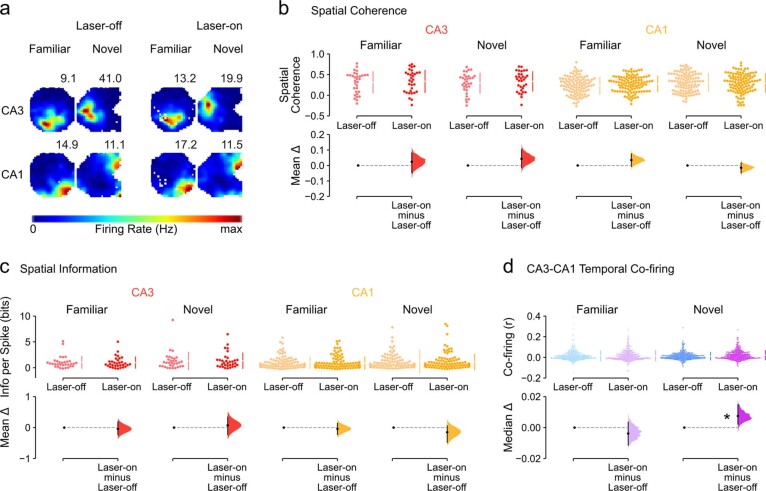
Silencing 4-7-week-old abDGCs preserves spatial information and coherence of CA3 and CA1 place maps but increases CA3-CA1 co-firing during spatial novelty. **(a)** Spatial rate maps from example CA3 and CA1 PCs (one cell per row) recorded from an abDGC::ArchT (4-7 wpi) mouse during laser-off and laser-on exploration sessions in the familiar and novel environments. The peak firing rate (Hz) is given above each map. **(b,c)** Silencing 4-7-week-old abDGCs during laser-on sessions did not affect the spatial coherence **(b)** or the spatial information **(c)** of CA3 or CA1 place maps (p > 0.09 for spatial coherence; p > 0.25 for spatial information, two-sided permutation tests; DG = 49, CA3 = 36, CA1 = 126 cells in 4 mice). **(d)** Silencing abDGCs significantly increased temporal correlation of theta-nested spike trains in pairs of CA3 and CA1 PCs. However, note that this abDGC silencing effect on CA3-CA1 ensemble co-firing was seen during novel environment exploration (p = 0.4 for familiar, p = 0.03 for novel, two-sided paired permutation tests; n = 4087 CA3-CA1 cell-pairs in 3 mice). Panels b-d show Cumming estimation plots as described in Extended Data Fig. 1e. *p < 0.05.

We thus asked whether adult-born granule cell modulation of population firing structure serves hippocampal processing of novel mnemonic stimuli. During silencing of abDGCs in their 4–7-week maturation period, we noted that the CA PCs maintained their spatial tuning and place representation (Extended Data Fig. 5a–c). But the hippocampus also represents its computations by the temporally structured, collective activity (co-firing) of its PCs, in addition to their individual firing tuning^1^. We thus calculated the strength of the pairwise co-firing associations between CA3 and CA1 PCs, using the Pearson correlation coefficient of their theta-nested spike trains, separately for laser-off and laser-on sessions in familiar versus novel environments. In fact, the network contained significantly more temporally correlated CA3–CA1 spike trains during abDGC silencing in novel environments, indicating reduced population sparsity in the absence of functional abDGCs (Extended Data Fig. 5d). This suggested that abDGCs promote enhanced sparsity of the population firing structure during novel stimulus processing, allowing co-existing neural patterns to lie in a higher-dimensional network activity space where their reduced overlap augments input discrimination and memory capacity.

**Fig. 6:**
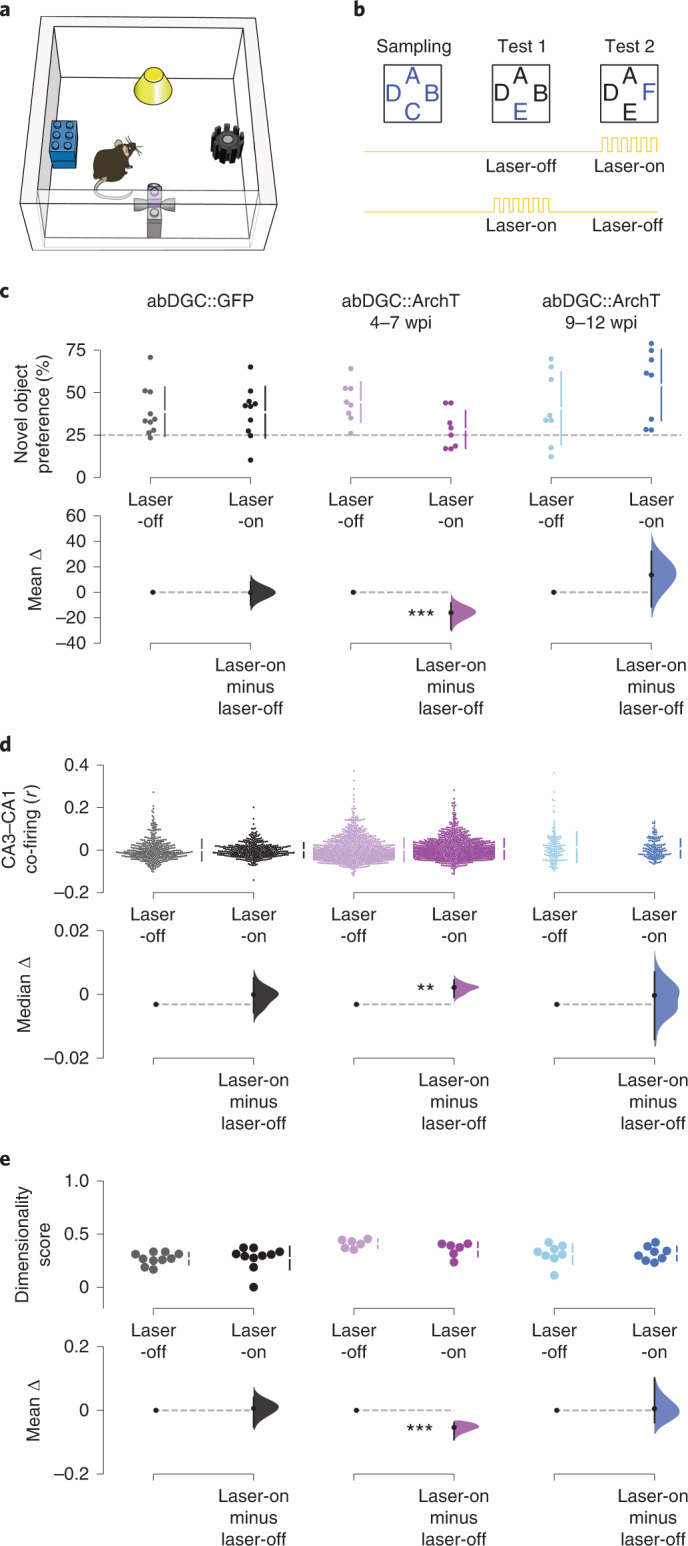
Silencing 4–7-week-old abDGCs impairs novel object preference, increases CA3–CA1 co-firing and reduces population dimensionality. **a**,**b**, cNOR task arena (**a**) and layout (**b**). Letters depict object locations (novel objects in blue). Each day, one laser-off alternated with one laser-on test to provide within-day comparison. **c**, Estimation plot showing the percentage of time spent by mice in the abDGC::GFP and abDGC::ArchT groups with novel objects in laser-off and laser-on tests. Upper plot: each data point represents the mean percentage time spent with the novel object; chance performance is shown by the dashed line. Lower plot: mean difference in novel object exploration time (%) for laser-on relative to laser-off tests. Mice exhibited significantly lower novel object preference during 4–7-week-old abDGC silencing (abDGC::GFP: *n* = 10 sessions in 2 mice; abDGC::ArchT (4–7 wpi): *n* = 8 sessions in 4 mice; abDGC::ArchT (9–12 wpi): *n* = 8 sessions in 2 mice). **d**, CA3–CA1 ensemble co-firing. Upper panel: each data point represents the correlated activity of one pair of simultaneously recorded CA3–CA1 PCs. Lower plot: median difference between co-firing in laser-on versus laser-off tests (abDGC::GFP: *n* = 400 CA3–CA1 cell pairs in 2 mice; abDGC::ArchT (4–7 wpi): *n* = 1,080 CA3–CA1 cell pairs in 3 mice; abDGC::ArchT (9–12 wpi): *n* = 150 CA3–CA1 cell pairs in 2 mice). **e**, Dimensionality of PC population theta co-firing vectors between laser-off and laser-on tests (dimensionality score = estimated dimensionality/number of PCs in each vector; abDGC::GFP: *n* = 10 sessions in 2 mice; abDGC::ArchT (4–7 wpi): *n* = 6 sessions in 3 mice; abDGC::ArchT (9–12 wpi): *n* = 8 sessions in 2 mice). Panels **c**–**e** show Cumming estimation plots as in Fig. 1f. ***P < 0.001, **P < 0.01.

**Extended Data Fig. 6:**
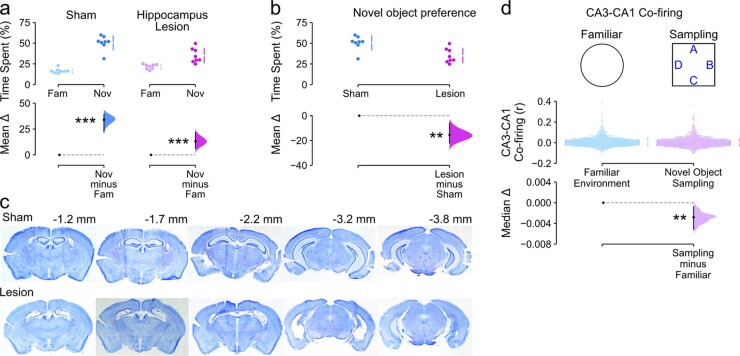
Hippocampal dependency of novelty preference and decorrelation of CA3-CA1 firing patterns in the continuous multi-object recognition task. **(a-c)** Hippocampal lesions impair novelty preference in the continuous multi-object recognition task. **(a)** Mice that received sham lesions (n = 8 mice) showed a strong behavioural preference for the novel object over the familiar objects (p = 0.001, two-sided paired permutation test). Mice with hippocampal lesions (n = 8 mice) showed a weaker but still significant novelty preference (p = 0.001, two-sided paired permutation test). **(b)** Direct comparison showed significantly stronger novelty preference in sham compared to hippocampal-lesioned mice (p = 0.005, two-sided permutation test). **(c)** Example histology of coronal brain sections from one sham-lesioned and one hippocampal lesioned mouse (with anterior-posterior position from bregma above each section). **(d)** Behavioural experience of novel objects is associated with reduced CA3-CA1 ensemble co-firing. Using ensemble recordings during behavioural sampling of novel objects (blue letters), we found that CA3-CA1 pair-wise firing correlations were significantly lower compared to those measured during exploration of a familiar (circular-walled) environment without objects (p = 0.002, two-sided paired permutation test, n = 1721 CA3-CA1 cell-pairs in 7 mice). Upper panel: each data point represents the correlated activity of one-pair of simultaneously recorded CA3-CA1 PCs. Lower plot: median difference between co-firing in novel object sampling minus familiar arena. Notably, this finding shows that processing of new information is naturally associated with a decorrelation of CA3-CA1 firing patterns. Panels a-b,e show Cumming estimation plots as described in Extended Data Fig. 1e. ***p < 0.001, **p < 0.01.

To test this hypothesis, we trained mice to continually process novelty during a 1-d multi-object recognition task. On each day, mice first explored a familiar arena (without objects) to monitor baseline network activity. Mice then repeatedly explored another, square-walled arena containing four objects (Fig. 6a). In this ‘object arena’, mice initially encountered four distinct novel objects, each one placed beside a wall (Fig. 6b; ‘Sampling’). On each subsequent session (Fig. 6b; ‘Tests’), one of the initially sampled objects was replaced with a different novel object so that the mouse could explore one completely novel object along with the three ‘familiar’ objects seen in the previous session that day. In these tests, we measured novelty detection using the proportion of time spent investigating the novel versus the familiar objects. Hippocampal lesions impaired behavioral performance in this task (Extended Data Fig. 6a–c). Moreover, using ensemble DG-CA3-CA1 recordings, we noted that the network exhibited significantly weaker CA3–CA1 co-firing during the sampling phase when mice explored the novel objects compared with the exploration session in the familiar arena (Extended Data Fig. 6d), indicating sparser population firing structure during hippocampal processing of novel information.

**Extended Data Fig. 7:**
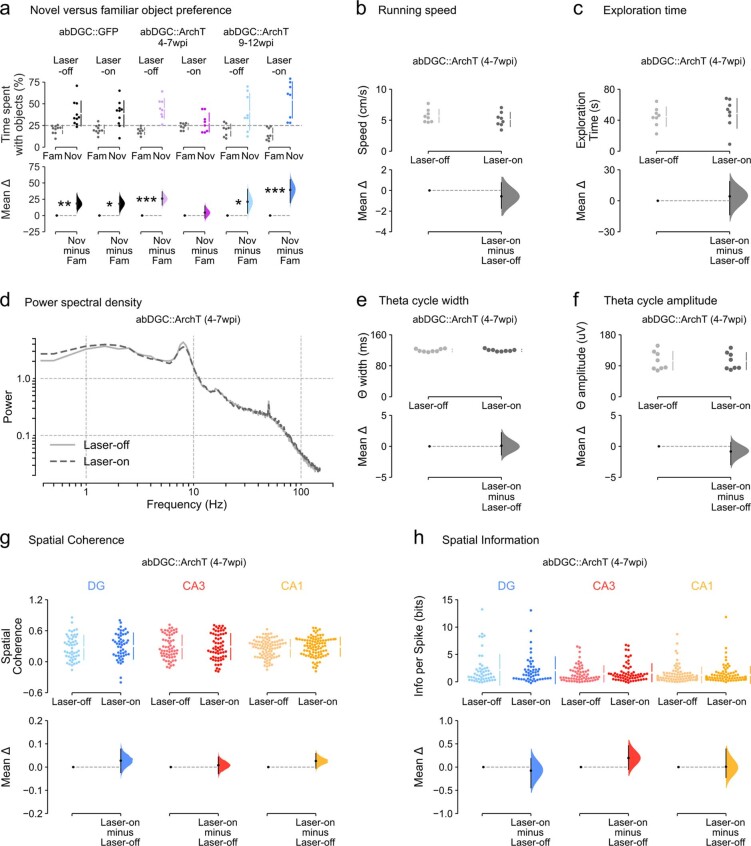
Silencing 4-7-week-old abDGCs impairs novelty preference but does not alter running speed, exploration time, theta oscillations, or spatial tuning of hippocampal maps during the multi-object recognition task. **(a)** Upper plot: each grey data point represents the mean time spent with the three familiar objects and each coloured data point represents the time spent with the novel object; chance performance is shown by the dashed line. Lower plot: mean difference between novel and familiar object and exploration time. All groups exhibited a preference for novel over familiar objects during laser-off tests (abDGC::GFP: p = 0.003, abDGC::ArchT (4-7wpi): p = 0.001; abDGC::ArchT (9-12wpi): p = 0.035, one-sided paired permutation tests). During laser-on tests, 4-7-week-old abDGC silencing resulted in an absence of novelty preference (p = 0.4), whereas novelty preference was maintained during laser-on tests in abDGC::GFP (p = 0.016) and abDGC::ArchT (9-12wpi) mice (p = 0.001, two-sided paired permutation tests; n = 10, 8, and 8 sessions in 2 abDGC::GFP, 4 abDGC::ArchT (4-7wpi), and 2 abDGC::ArchT (9-12wpi) mice, respectively). **(b,c)** In abDGC::ArchT mice, silencing 4-7-week-old abDGCs during the continuous novel object recognition task did not affect running speed (**b**, p = 0.4) or exploration time (**c**, p = 0.6, two-sided permutation tests from 8 sessions in 4 mice). **(d-f)** Silencing 4-7-week-old abDGCs did not also change single-cycle width (**e**, p = 0.9) or amplitude (**f**, p = 0.5) of theta oscillations recorded in the CA1 local field potentials (two-sided paired permutation tests, uncorrected for multiple comparisons, from 8 sessions in 4 mice). **(g)** Spatial coherence **(h)** and spatial information **(e)** in DG, CA3 and CA1 PCs were also unaffected during 4-7-week-old abDGC silencing during the task (p > 0.1 and p > 0.1, respectively, two-sided paired permutation tests from DG = 51, CA3 = 63, CA1 = 91 cells in 4 mice). Panels a-c and e-h show Cumming estimation plots as described in Extended Data Fig. 1e. ***p < 0.001, **p < 0.01, *p < 0.05.

In both abDGC::ArchT and abDGC::GFP mice, we applied DG light delivery on either the first or second test on alternate days (Fig. 6b), so that each day provided one laser-on versus one laser-off test to allow counter-balanced, within-day comparison. In all groups, mice exhibited a significant preference for the novel over the familiar objects during laser-off tests (Extended Data Fig. 7a; novel versus familiar objects, *P* < 0.05, paired permutation tests). In both abDGC::GFP and 9–12-wpi abDGC::ArchT groups, mice continued to express this novelty preference during laser-on tests (Fig. 6c and Extended Data Fig. 7a). However, silencing abDGCs during the 4–7-wpi period significantly impaired novel object recognition in abDGC::ArchT mice (laser-off versus laser-on tests: *P* < 0.001, paired permutation test), reducing it to chance level (Fig. 6c and Extended Data Fig. 7a). Silencing 4–7-week-old abDGCs did not alter total exploration time or running speed, nor did it alter theta amplitude or frequency, or spatial tuning of the other DG, CA3 and CA1 PCs (Extended Data Fig. 7b–h).

To identify the network operations requiring abDGCs in this task, we quantified theta-paced neuronal co-firing between CA3 and CA1 PCs, directly comparing laser-off tests with laser-on tests. Neither silencing 9–12-week-old abDGCs in abDGC::ArchT mice nor light delivery in abDGC::GFP mice affected CA3–CA1 co-firing in laser-on tests compared with laser-off tests (Fig. 6d; *P* > 0.2, paired permutation tests). However, silencing 4–7-week-old abDGCs significantly increased CA3–CA1 co-firing in laser-on tests (Fig. 6d; *P* = 0.0028, paired permutation test). These results suggest that network decorrelation of CA3–CA1 theta co-firing patterns involves 4–7-week-old abDGCs during behavioral discrimination of novel versus familiar stimuli.

**Extended Data Fig. 8:**
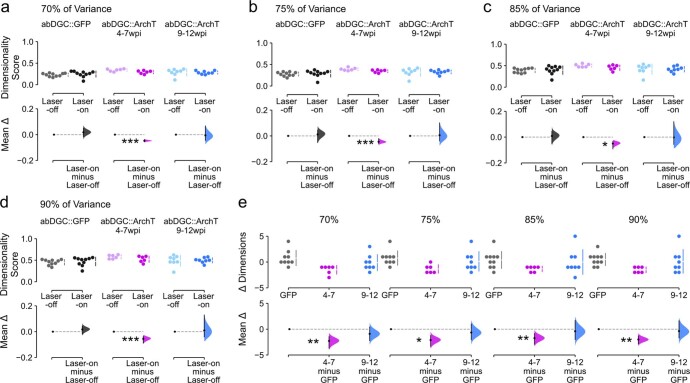
Principal component analysis of hippocampal population dimensionality for various levels of explained variance. To corroborate the results we obtained on the dimensionality of principal cell (PC) population theta co-firing vectors in laser-off and laser on tests in the cNOR task (Fig. 6e), we repeated the principal component analysis (PCA) for different proportions of explained variance. In Fig. 6e we show the number of dimensions required to explain 80% of the variance, with each data point scaled by the number of PCs in each vector. **(a-d)** Panels a-d show the same analysis but for 70%, 75%, 85% and 90%, respectively. Only abDGC::ArchT (4-7wpi) mice exhibit reduced dimensionality during laser-on versus laser-off tests (p = 0.001, p = 0.001, p = 0.03, p = 0.001, respectively, two-sided paired permutation tests, n = 10, 6, and 8 sessions in 2 abDGC::GFP, 3 abDGC::ArchT (4-7wpi), and 2 abDGC::ArchT (9-12wpi) mice, respectively). **(e)** The corresponding absolute change in dimensionality (laser-on minus laser-off) for the three groups of mice at different proportions of explained variance as in a-d. In each case, abDGC::ArchT (4-7wpi), but not abDGC::ArchT (9-12wpi) were significantly lower than the abDGC::GFP group (p = 0.005, p = 0.02, p = 0.01, p = 0.01, respectively, two-sided permutation tests). The mean number of PCs in each vector was as follows: abDGC::GFP 22.7 ± 2.2; abDGC::ArchT (4-7wpi) 31.7 ± 5.1; abDGC::ArchT (9-12wpi) 27.0 ± 4.9. There were no group differences in the number of PCs in each vector (F(2,21) = 1.1, p = 0.4; one-way ANOVA). Panels a-e show Cumming estimation plots as described in Extended Data Fig. 1e. ***p < 0.001, **p < 0.01, *p < 0.05.

Effective discriminative responses in cortical circuits entail a high-dimensional population firing structure^28^. We thus determined whether abDGCs enable higher-dimensional hippocampal activity during our novel object task. To evaluate this, we generated population activity matrices (PCs × theta cycles) using the PC spikes discharged in the theta cycles of laser-off and laser-on tests and then calculated the number of principal components explaining 80% of the variance across theta-nested population vectors in each test. Silencing 9–12-week-old abDGCs in abDGC::ArchT mice and light delivery in abDGC::GFP mice did not affect population dimensionality for laser-on versus laser-off tests (Fig. 6e). However, silencing 4–7-week-old abDGCs decreased the number of principal components in laser-on compared with laser-off tests, showing lower-dimensional population firing structure when novel object recognition was impaired (Fig. 6e; *P* = 0.001, paired permutation test; Extended Data Fig. 8). This occurred while both theta cycle duration and amplitude remained unchanged by optogenetic silencing (Extended Data Fig. 7e,f). Thus, abDGCs enable a higher-dimensional population firing structure during successful novelty detection.

## Discussion

Our findings identify an age-dependent role for adult-born DG neurons in promoting higher-dimensional firing structure of the hippocampal network where patterns of sparse population activity serve memory. Sparsening activity patterns constitutes an important operation in the nervous system to optimize capacity and reduce interference (cross-talk) between co-existing representations through decorrelation (orthogonalization)^25,29,30^.

In this study, we first observe that abDGCs constitute a set of high-firing, strongly theta-modulated granule cells. Early single-unit electrophysiological work and more recent calcium imaging work report strikingly low firing rates in DG granule cells, several-fold lower than CA3 and CA1 PCs^15,31^. Using triple-(DG-CA3-CA1) ensemble recordings during exploratory behavior, we found that abDGCs are not only more active than other PCs recorded in DG but also than those recorded in CA3 and CA1. This elevated firing rate of tetrode-recorded abDGCs is in line with work showing higher activity of abDGCs compared with mature DG cells using two-photon imaging of running-related calcium transients^32^. The exact magnitude of the activity difference between abDGCs and other PCs remains for future work to address since multichannel electrophysiology and calcium imaging assess activity rates and firing patterns differently. Nevertheless, the division of the DG population into higher- versus lower-rate granule cells provides an intriguing parallel to recent work showing two subpopulations of high- and low-firing CA1 pyramidal cells, which exhibit differential response to spatial novelty and learning^33,34^. However, high-rate CA1 PCs are born earlier than low-rate CA1 PCs during hippocampus development^35^, opposite to the situation found with developmental versus adult-born DG granule cells. The higher firing activity of abDGCs could relate to their enhanced response to afferent input, lower input specificity or greater synaptic plasticity^18–20,32,36,37^ but may also reflect reduced perisomatic inhibition or other properties^18–20,32,36–39^. Compared with mature granule cells, young abDGCs preferentially receive inputs from the lateral over the medial entorhinal cortex^8,40^. With the lateral entorhinal cortex central to novel information recognition^41^, abDGCs are thus well placed to influence hippocampal firing dynamics during novelty detection^42^. Consistent with this, we observed that abDGCs’ high spiking rate further increases as a natural response to novelty, which we found associated with reduced CA3–CA1 co-firing.

Similar to other hippocampal PCs, abDGCs could support memory by acting as information coding units for internal representation of experienced variables^43–45^. Of particular relevance, decades of work support a key role for hippocampal place cells in memory. However, compared with the other DG PCs, we found that abDGCs exhibit low spatial tuning, although this improves with their maturation. The weaker spatial tuning in abDGCs versus their mature DG counterparts is also in line with observations reported by Danielson and colleagues using calcium imaging^32^. However, abDGCs could also assist hippocampus-wide dynamics in addition to being representational building blocks. For instance, abDGCs could influence activity beyond the local DG circuitry via GABAergic interneurons^21,38,46–48^ or within the local circuitry via interneurons or other granule cells^5,48,49^. In line with this, previous in vitro work has shown that, by 4 weeks post-birth, abDGCs can promote excitation and feed-forward inhibition onto CA3 PCs, further suggesting that after 7 weeks of age, abDGCs can then reliably recruit inhibition onto mature DG granule cells^21^. Other studies yet report that young (<6 weeks old) abDGCs can also influence activity of mature DG granule cells^5,49^. Moreover, compared with 7-week-old abDGCs, 4-week-old abDGCs receive weaker lateral inhibition following activation of mature DG granule cells^21^. These findings are not only consistent with a functional contribution of abDGCs to hippocampus-wide dynamics, but importantly with one that continues to evolve over time. Here, our data support a time-varying modulatory role for abDGCs by revealing in vivo the network-level consequences of their manipulation during the time windows of 4–7 versus 9–12 weeks of maturation.

We started by observing that activating abDGCs influences CA3–CA1 ensemble spiking along with feed-forward inhibition, thereby promoting sets of instantaneous population vectors where PC spiking activities are sparsely nested in the timeframe of individual theta cycles. However, this sparsification operation appears to be limited to a ~4–7-week critical period of abDGC maturation. Manipulating abDGCs thereafter, at 9–12 weeks of age, no longer influences CA population sparsity. Previous in vitro and in vivo anaesthetized rodent studies have reported that activation of DG PCs can lead to net inhibition of individual CA3 PCs, suggesting that sparsening downstream activity could be a general feature of all DG PCs^50–52^. Here, we found that activating abDGCs did not produce a net change in the average CA3 population firing. Moreover, both silencing 9–12-week-old abDGCs and silencing a larger fraction of DG PCs (in Grm2^DG^::ArchT mice) did not change average CA3 PC firing or population sparsity. In contrast, silencing 4–7-week-old abDGCs produced a marked increase in CA3 and CA1 population firing and decreased sparsity. This age-dependent effect of abDGCs on the structure of network activity could reflect a change in their connectivity to selective postsynaptic targets. For example, abDGC wiring could transiently utilize the heterogeneity of GABAergic interneuron populations where diverse members provide coordinated rhythmic inhibition to distinct principal subcellular compartments^53^. This could further leverage the heterogeneity of the CA3 pyramidal cell population where, for instance, thorny versus athorny members differentially promote CA1 synchronicity^54^. The maturation-dependent network contribution of abDGCs might also involve a change in synaptic signaling since glutamatergic and GABAergic forms of transmission have been suggested to coexist at mossy fibers^55,56^.

Our findings reveal that abDGCs exert a network-level modulatory role throughout their maturation, but this role evolves over time. Young (4–7 weeks) abDGCs act beyond the local DG circuitry, influencing theta-nested CA population patterns. This effect on CA3–CA1 sparsity is not simply replicated by activating another set of DG cells recruited irrespective of their birthdate. At 4–7 weeks old, abDGCs also suppress the activity of DG PCs, but when older (9–12 weeks), abDGCs can then entrain DG PCs. Thus, mature abDGCs do not reproduce the local circuit wiring sculpted during development, in line with recent work showing that mature abDGCs exhibit distinct morphological features compared with developmentally born neurons^57,58^. Together with work showing that the temporal origin of developmental DG cells determines single-neuron properties^59,60^, these findings converge to the view that the adult DG hosts a heterogeneous population of granule cells^61^. This heterogeneity could be central to the network’s ability to diversify population responses and support hippocampal functions. In this way, DG assemblies comprising members of distinct granule cell subpopulations may serve to adjust sparsity in downstream CA populations, allowing low- to high-dimensional activity patterns to meet ongoing task demands (for example, lower-dimensional and correlated for robust encoding/retrieval, higher-dimensional and uncorrelated for flexible encoding/retrieval)^28^. In line with this, silencing immature abDGCs reduces CA population dimensionality and impairs behavioral performance in our hippocampus-dependent, multi-object discrimination task. The absolute magnitude of such dimensionality changes would need to be determined in future experiments, since, unlike immediate early genes and calcium imaging, multichannel recordings inherently cannot detect the fraction of silent neurons that contribute to sparse population coding. Collectively, our findings provide perspectives for future studies to assess how ongoing changes in the adult DG circuitry and hippocampal population firing structure allow the neural computations and codes that underpin memory-guided behaviors.

## Methods

### Animals

To deliver light-sensitive proteins into abDGCs, these experiments used a Cre-LoxP approach in two different mouse models: (1) adult wild-type male C57Bl6/J mice injected with a retrovirus (Moloney Murine Leukemia Virus (MMLV) carrying Cre-recombinase; see details below) and an AAV (carrying Cre-dependent opsins) into the DG and (2) adult Nestin-Cre male mice injected with an AAV carrying Cre-dependent opsins into the DG. Nestin is an intermediate filament protein that is present in the neural progenitor cells that develop into abDGCs^27,62^. MMLVs deliver genes into the offspring of dividing cells, thus transfecting newborn cells^63^. Male hemizygous Nestin-Cre mice (Jackson Laboratories; B6.Cg-Tg(Nes-Cre)1Kln/J, stock no. 003771 (ref. ^64^), RRID: IMSR_JAX:003771) were crossed with female C57Bl6/J mice (Charles River Laboratories). The MMLV approach was used for both abDGC optogenetic ChR2 activation and ArchT silencing throughout this study. The Nestin-Cre approach was only used in the optogenetic ChR2-tagging experiments (Figs. 1 and 2 and Extended Data Figs. 1 and 2).

To optogenetically target another subpopulation of DG cells selected independently of their birthdate, we used adult c-fos–tTA transgenic male mice heterozygous for the transgene carrying the c-fos promoter-driven tTA^65,66^. This c-fos–tTA mouse line was generated at The Scripps Research Institute and maintained at Tufts University until shipment to the Medical Research Council (MRC) Brain Network Dynamics Unit (BNDU) at the University of Oxford. Mice were bred from c-fos–tTA mice crossed with C57Bl6/J mice.

To optogenetically target whole DG granule cells, we used adult metabotropic-glutamate-receptor 2-Cre (Grm2-Cre) hemizygous male mice. This Grm2-Cre mouse strain was obtained from the Mutant Mouse Resource and Research Center (MMRRC; Tg(Grm2-cre)MR90Gsat/Mmucd; stock no. 034611-UCD, RRID: MMRRC_034611-UCD) at University of California at Davis, a National Institutes of Health (NIH)-funded strain repository, and was donated to the MMRRC by Nathaniel Heintz, Ph.D., The Rockefeller University, GENSAT and Charles Gerfen, Ph.D., NIH, National Institute of Mental Health.

For the hippocampal lesion experiments, we used adult male wild-type C57Bl6/J mice.

Mice were group-housed with same-sex littermates until the start of the experiment; microdrive-implanted mice were singly housed after surgery. The age of the mice used in this study ranged from 4 to 6 months. Mice had free access to food and water throughout in a dedicated housing room with a 12/12-hour light/dark cycle (7:00 to 19:00), 19–23 °C ambient temperature and 40–70% humidity. All experiments were performed between 8:00 and 18:00. The numbers of mice recorded in each treatment group are given in Supplementary Table 1. Experiments were performed on mice in accordance with the Animals (Scientific Procedures) Act, 1986 (United Kingdom), with final ethical review by the Animals in Science Regulation Unit of the UK Home Office.

### Viral vectors

AAVs carrying double-floxed inverse open reading frame (DIO) Cre-dependent opsins were used to deliver ChR2 (ref. ^67^) under the Ef1a promoter, or ArchT^68^ under the CAG promoter, into the DG of adult mice (AAV9-EF1a-DIO-hChR2(E123T/T159C)-EYFP-WPRE, titer: 2 × 10^12^ Transducing Units (TU) ml^−1^, University of Pennsylvania; AAV9-CAG-Flex-ArchT-GFP, titer: 8.3 × 10^12^ TU ml^−1^, University of North Carolina). To target abDGCs, wild-type C57Bl6/J mice were injected with both a retrovirus (MMLV-pMX-T2A-Cre-mCherry, Creative Biogene) to deliver Cre into abDGCs, and one of the aforementioned AAVs (to deliver either ChR2 or ArchT) in a 1:1 ratio. Retrovirus titer was 1.5 × 10^9^ Infectious Units (IFU) ml^−1^, and pRubiC-T2A-Cre was a gift from Bryan Luikart^63^ (Addgene plasmid no. 66692; http://n2t.net/addgene:66692; RRID: Addgene_66692). In additional experiments, abDGCs were also targeted in Nestin-Cre mice injected with the AAV carrying ChR2. To optogenetically activate another subpopulation of DG cells ChR2-targeted independently of their birthdate, we injected the DG of c-fos–tTA mice with a TRE3G-Cre AAV carrying the Cre-recombinase under the control of the third generation of tetracycline-responsive element containing promoter (TRE3G, Clontech Laboratories), which we mixed in a 1:5 ratio with the AAV carrying ChR2 used to target abDGCs in abDGC::ChR2 mice. For this, we generated a pAAV-TRE3G-FLAG-Cre plasmid by exchanging the ArchT-GFP-encoding fragment in the pAAV-TRE3G-ArchT-GFP vector^66^ with the fragment encoding the Cre-recombinase open reading frame by use of the EcoRI and NcoI restriction sites. The recombinase open reading frame was amplified with the primers 5′-GTTTCTGCCACCATGGATTACAAGGATGACGATGACAAGTTGGCCAATTTACTGACCG-3′ and 5′-GTTTCTGAATTCTCAATCGCCATCTTCCAGCAG-3′ using pCAG-Cre plasmid DNA as template. pCAG-Cre was a gift from Connie Cepko (Addgene plasmid no. 13775; http://n2t.net/addgene:13775; RRID: Addgene_13775)^69^. To optogenetically silence whole DG granule cells, we injected the DG of adult Grm2-Cre mice with the ArchT-GFP AAV otherwise used to target abDGCs in abDGC::ArchT mice.

### Surgical procedures

Mice received viral injections and microdrive implantations under gaseous isoflurane anesthesia (~1% in 1 l min^−1^ O_2_), with systemic and local analgesia administered subcutaneously (meloxicam 5 mg kg^−1^; buprenorphine 0.1 mg kg^−1^; bupivacaine 2 mg kg^−1^). Viruses were injected bilaterally into the dorsal DG (3 × 200 nl each hemisphere in abDGC::ChR2, abDGC::ArchT, abDGC::GFP and Grm2^DG^::ArchT mice using the stereotaxic coordinates from bregma: anterior–posterior: −1.6, −2.4, −2.4; medio–lateral: ±1.0, ±1.2, ±1.5; dorso–ventral: −1.7, −1.7, −1.7, respectively; 2 × 25 nl in c-fos^DG^::ChR2 mice using the stereotaxic coordinates from bregma: anterior–posterior: −1.6, −2.4; medio–lateral: ±1.0, ±1.5; dorso–ventral: −1.7, −1.7, respectively). Viruses were delivered using a pulled glass micropipette (~16-µm internal diameter) at a rate of 100 nl min^−1^, with an additional 100 nl min^−1^ of diffusion time with the pipette in situ. At 2 weeks after virus injection, mice were implanted with a microdrive^70^ containing 12 or 14 independently movable tetrodes bilaterally targeting DG, CA3 and CA1, and two optic fibers (Doric Lenses) positioned bilaterally above the dorsal DG. Electrophysiological recordings began ~2 weeks after microdrive implantation, and transfected abDGCs were ~4–7 or 9–12 weeks old at the time of recording. For the hippocampal lesion surgery, mice in the lesion group (*n* = 8) underwent the same anesthetic induction as above, then scalp incision and craniotomy, followed by NMDA (10 mg ml^−1^) injections directly into the hippocampus at four sites per hemisphere using a modified Hamilton 36-G syringe needle (anterior–posterior: −1.7, −2.3, −2.8, −3.1; medio–lateral: ±1.2, ±1.7, ±2.2, ±2.8; dorso–ventral: −1.9, −1.9, −2.0, -4.0, respectively, 100–200 nl per site at the infusion/diffusion rates described above), and were then sutured. Midazolam (5 mg kg^−1^, subcutaneous) was used to prevent seizures in hippocampal-lesioned mice. Mice receiving sham surgery (*n* = 8) were incised and then sutured. All mice had at least 2 weeks of recovery before behavioral testing.

### Recording procedures

Following microdrive implantation surgery, mice recovered for at least 7 days before familiarization to the recording procedure. Mice were handled daily and exposed to the familiar environment and sleep box for >0.5 hours per day for at least 4 days. During this period, tetrodes were slowly lowered to the proximity of the cell layers. For all mice, on the morning of each recording day, tetrodes were lowered into the CA1, CA3 pyramidal or DG granule cell layers in search of multi-unit spiking activity, using the electrophysiological profile of the LFPs including sharp-wave ripples, gamma oscillations and dentate spikes to further guide placement. Tetrodes were left in position for ~1.5–2 hours before recordings started that day. At the end of each recording day, tetrodes were raised (~150 µm) to avoid damaging the cell layers overnight. During recording sessions, mice explored familiar and novel environments (41-cm-diameter cylinder, or 41 × 41-cm^2^ square box, both with 30-cm-high walls) or were placed in a 12 × 12 × 28 (height)-cm^3^ sleep box. A white cue card was placed on the wall of one side of the familiar environment. Wooden inserts were placed into the square environment each day to create different novel geometric configurations. The centroid positions of the familiar and novel environments were in the same location within the recording room. The sequence of familiar and novel environment exposure varied on each day. Each recording session (including the opto-tagging sessions) lasted ~15 min. Experiments were performed under dim-light conditions (~20 lux) with low-level background noise (~50 dB).

### Dox treatment

Food containing Dox (‘regular Dox’; 40-mg kg^−1^ chow pellets; Bio-Serv) was provided to c-fos–tTA mice for at least 2 weeks before the injection surgery. Following recovery after the microdrive implantation, the familiarization to the recording procedure described above was temporarily discontinued for c-fos^DG^::ChR2 mice to allow activity-dependent ChR2-tagging. That is, c-fos^DG^::ChR2 mice were taken off the regular Dox 48 hours before being exposed to an open-field enclosure for 30 min. Immediately after this short tagging procedure, c-fos^DG^::ChR2 mice went back to their home cage and were provided with a high dose of Dox^66^ for 24 hours, subsequently returning to regular Dox diet the following day and for the remainder of the experiment. The c-fos-dependent labeling was thus driven by a 30-min exploration of an open field.

### Light delivery

We used 561-nm and 473-nm diode-pumped solid-state lasers (Crystal Laser, models CL561-100 and CL473-100; distributer: Laser 2000) to deliver light bilaterally to the dorsal DG (~5–9 mW) via a two-channel rotary joint (Doric Lenses). For both optogenetic identification of abDGCs (abDGC::ChR2 mice) and assessing changes in CA population sparsity (abDGC::ChR2 and c-fos^DG^::ChR2 mice), we used 5-ms blue-light pulses, delivered either with a random uniformly distributed inter-stimulus interval (0.5 to 2.5 s) in open-field environments or at 0.3 Hz (when in the sleep box). Using yellow-light delivery, abDGC::ArchT, abDGC::GFP and Grm2^DG^::ArchT mice received 30-s-duration pulses, with a random uniformly distributed inter-stimulus interval (range: 5 to 22 s).

### Multichannel data acquisition

Electrode signals were amplified, multiplexed and digitized using a single integrated circuit (headstage) located on the head of the animal (RHD2164, Intan Technologies; http://intantech.com/products_RHD2000.html). The amplified and filtered (pass band 0.09 Hz to 7.60 kHz) electrophysiological signals were digitized at 20 kHz (RHD2000 Evaluation Board) and saved to disk with the synchronization signals from the positional tracking and laser activation. To track the location of the animal, three LEDs were attached to the headstage and captured at 25 frames per second by an overhead color camera.

### Spike sorting and unit isolation

Spike sorting and unit isolation were performed via automatic clustering software Kilosort^71^ (https://github.com/cortex-lab/KiloSort), followed by graphically based manual recombination using cross-channel spike waveforms, auto-correlation histograms and cross-correlation histograms within the SpikeForest framework (https://github.com/flatironinstitute/spikeforest)^72^. All sessions recorded on a given day were concatenated and cluster-cut together to monitor cells throughout the day. Units that were well isolated and stable over the entire recording were used for analysis. Hippocampal PCs and interneurons were identified by their auto-correlograms, firing rates and spike waveforms as described previously^73^. In total, this study includes *n* = 5,158 hippocampal PCs (abDGC::ChR2 (4–7 wpi): CA1 *n* = 748, CA3 *n* = 201, DG *n* = 887, abDGC *n* = 33, from 8 mice; abDGC::ChR2 (9–12 wpi): CA1 *n* = 463, CA3 *n* = 102, DG *n* = 160, from 3 mice; c-fos^DG:^:ChR2: CA1 *n* = 385, CA3 *n* = 12, DG *n* = 7, from 3 mice; abDGC::ArchT (4–7 wpi): CA1 *n* = 490, CA3 *n* = 347, DG *n* = 230, abDGC *n* = 13, from 4 mice; abDGC::GFP mice: CA1 *n* = 124, CA3 *n* = 71, DG *n* = 147, from 2 mice; abDGC::ArchT (9–12 wpi): CA1 *n* = 206, CA3 *n* = 55, DG *n* = 108, from 2 mice; Grm2^DG:^:ArchT: CA1 *n* = 282, CA3 *n* = 92, DG *n* = 272, from 4 mice). Further details can be found in Supplementary Table 2.

### Identification of light-modulated units

In abDGC::ChR2 mice, firing responses to light delivery were characterized by calculating, for each neuron, the spike discharge across 1-ms time bins relative to laser-onset. For a cell to be classified as an abDGC, a light-driven increase in *Z*-scored firing rate had to occur within 10 ms of laser-onset and be >3 s.d. above baseline (calculated from a 200-ms epoch before laser-onset). This way, we identified 33 abDGCs out of 920 DG PCs (3.6%) in abDGC::ChR2 mice (mean firing response latency = 3.7 ± 0.5 ms). We further determined the false discovery rate (FDR) by using the same classification criterion on the shuffled spike trains of each neuron and found only 1 out of 920 DG PCs that met this criterion (FDR: ~0.1%).

In addition, we observed that 5-ms laser pulses evoked a delayed (~15–40-ms) increase in spiking activity of some DG, CA3 and CA1 interneurons in abDGC::Chr2 (4–7 wpi) mice. For display purposes, we present peristimulus time histogram (PSTH) examples from individual interneurons of the *Z*-scored change in firing rate (Fig. 2f), expressed in s.d. units. Respectively, 4%, 11% and 8% of DG, CA3 and CA1 interneurons in abDGC::ChR2 mice showed delayed light-modulation following abDGC optogenetic activation during the 4–7-week period following viral injection.

In abDGC::ArchT mice, for a cell to be classified as a putative abDGC, the light-driven *Z*-scored reduction in firing rate had to be >2 s.d. below baseline (calculated from a 4-s epoch before laser-onset) and present in at least 60% of time bins (800-ms bin width) during the 16 s after laser-onset. The different criteria used for classifying abDGCs during optogenetic silencing in abDGC::ArchT mice versus activation in abDGC::ChR2 mice reflect the fact that detecting a significant decrease in firing from a low baseline takes longer than detecting an increase in firing. We identified 13 of 243 (5.3%) abDGCs in abDGC::ArchT mice at 4–7 wpi and 5 of 120 abDGCs (4.2%) in abDGC::ArchT mice at 9–12 wpi (there was no difference between these percentages: *P* = 0.6). Note that none of the neurons recorded in abDGC::ArchT mice were included in the analyses presented in Figs. 1 and 2 concerned with the network effect of abDGC activation. In addition, in abDGC::ArchT (4–7 wpi) mice we identified a subset of interneurons that significantly reduced their firing rate during abDGC optogenetic silencing. The criterion used to identify these interneurons was a *Z*-scored reduction in firing rate >2 s.d. below baseline (calculated from a 4-s epoch before laser-onset), present in at least 40% of time bins during the first 16 s after laser-onset. The proportions of light-modulated DG, CA3 and CA1 interneurons in abDGC::ArchT (4–7 wpi) are given in Extended Data Fig. 4f.

### Estimating age of abDGCs

The age of the identified abDGCs was estimated using the number of days elapsed between virus injection and the recording day in which the abDGC was recorded.

### Place maps

To generate place maps, we divided the horizontal plane of the recording enclosure into spatial bins of 1.4 × 1.4 cm^2^ to generate the spike count map (number of spikes fired in each bin) for each neuron and the occupancy map (time spent by the animal in each spatial bin) in each task session. All maps were then smoothed by convolution with a two-dimensional Gaussian kernel (s.d. = 1.2 bin widths). Finally, spatial rate maps were generated by normalizing the smoothed spike count maps by the smoothed occupancy map. Spatial coherence (that is, the similarity of a cell’s firing rate in a given spatial bin over the firing rates in adjacent bins) was calculated from the unsmoothed place maps as the Pearson correlation coefficient between the firing rate in each spatial bin versus the mean firing rate in the eight neighboring spatial bins.

### Spatial information

The amount of spatial information conveyed by the spike train of a given cell was calculated using the formula proposed previously^74^:

$${\mathrm{Information}}\,{\mathrm{per}}\,{\mathrm{spike}} = \mathop {\sum}\limits_{i = 1}^N {P_i} \frac{{\lambda _i}}{\lambda }{\mathrm{log}}_2\frac{{\lambda _i}}{\lambda }$$

where *i* = 1, 2, …, *N* represents each spatial bin of the environment; _*Pi*_ is the probability of occupancy of bin *i*; *λ*_*i*_ is the mean firing rate in bin *i*; and *λ* is the mean firing rate of the cell over all spatial bins. We estimated spatial information for three different spatial bin sizes: ~1.4 cm (Fig. 1h), ~2 cm (Extended Data Fig. 2c) and ~4 cm (Extended Data Fig. 2d), by dividing the 41-cm arena into 30 × 30, 20 × 20 and 10 × 10 bins, respectively. We also computed a null distribution by shuffling spike times with respect to the location (Extended Data Fig. 2b).

### Theta-coupling analysis

Raw LFPs were downsampled from 20 kHz to 1,250 Hz (order 8 Chebyshev type I filter was applied before decimation to avoid aliasing) and then decomposed using Empirical Mode Decomposition (https://pypi.org/project/emd/)^75^. We determined individual theta cycles and theta phase from the CA1 LFP^70^. Briefly, we first detected peaks and troughs of theta with absolute values higher than the low-frequency component (sum of all components with main frequencies below the theta signal) envelope, and then a theta cycle was defined by pairs of supra-threshold troughs separated at least by 71 ms (~14 Hz) and no more than 200 ms (5 Hz) that surrounded a supra-threshold peak. Theta phase was calculated by interpolation through neighboring theta troughs, zero crossings and peaks. In abDGC::ChR2 mice, to analyze theta-coupling of abDGCs and DG, CA3 and CA1 PCs, we used only recording days that had at least one opto-tagged abDGC. For each neuron, we calculated the mean preferred firing phase and the spike-phase coherence, quantified as the mean resultant vector length. For all analyses of mean preferred phase and spike-phase coherence, spikes from all PCs were included, independent of whether they were significantly modulated by theta phase.

### Gamma coupling analysis

Epochs of slow gamma activity were detected using a band-pass filter (30–55 Hz) on the DG LFP (Extended Data Fig. 3a). Inclusion criteria were (1) presence of slow gamma activity in the DG LFP, >2 s.d. above the amplitude envelope; and (2) that the neuron fired at least 200 spikes during slow gamma epochs. Because gamma oscillations are local to a particular subfield, we compared the phase and depth of modulation of abDGCs with other DG PCs but not with CA3 or CA1 PCs.

### Spontaneous spiking cross-correlation analysis

We computed the discharge probability of hippocampal PCs and interneurons with respect to the spontaneous discharge of abDGCs (that is, in the absence of any optogenetic activation; Fig. 2e, observed spikes). Using recording days that had at least one opto-tagged abDGC, we computed cell-pair cross-correlations with each abDGC as the reference cell and all other simultaneously recorded neurons as the target cells. The cross-correlation for each target cell was computed as a conditional probability, *P*(*target cell spike* | *time*), where time zero indicates the spike times for the reference (abDGC) cell. Cross-correlations were generated between −160 ms and +160 ms, with a bin width of 0.8 ms. For display purposes, all cross-correlograms for a given cell type were averaged to produce group means (Fig. 2e, black histograms between −160 and +160 ms). To disentangle the contribution of theta modulation of all hippocampal spikes, we also computed control cross-correlations by shifting each observed spike to a random theta cycle while preserving its original theta phase^70^ (Fig. 2e). Therefore, shuffled spike trains had the same theta phase distribution as the spontaneously observed spikes. For each neuron we generated 500 surrogate distributions and generated an average distribution per neuron from the mean value at each time bin. Finally, we subtracted the shuffled spike distribution from the observed spikes to produce a difference distribution (Fig. 2e, black histograms between −20 and +60 ms). We performed an identical analysis with high-firing-rate DG PCs as the reference cells, by matching each DG PC’s firing rate to that of an abDGC taken from the same recording day (Extended Data Fig. 3d).

### Population-level sparsity of PC firing patterns

The sparsity *S* of a given population firing vector *x* was calculated using the Gini index^76–78^ as:

$$S = \frac{{\mathop {\sum}\nolimits_{i = 1}^N {(2i - N - 1)x_i} }}{{N\mathop {\sum}\nolimits_{i = 1}^N {x_i} }}$$

where *x* is the population vector containing, in ascending order, the spike counts discharged by each PC in a ~theta-cycle-long (100-ms) time window; *N* is the length of that vector (that is, the number of simultaneously recorded PCs); and *i* is the rank of spike counts in ascending order. Population vectors where the total number of spikes is more evenly distributed between neurons have a lower Gini index (lower sparsity) than population vectors where the total number of spikes is concentrated in a few neurons (higher sparsity). To investigate the consequence of abDGC or other DG activation on hippocampal population sparsity (Figs. 2g, 3c and 4c), we used the 100-ms time bins before versus the 100-ms time bins after blue-laser-onset. Gini indices were computed for every laser pulse on a given recording session and then averaged to get one pair of ‘No activation’ and ‘abDGC activation’ values (or equivalent) for each session (Fig. 2g). This within-session, paired analysis controlled for differences in the number of simultaneously recorded PCs across different recording days^77^. To investigate the consequence of abDGC or other DG silencing on hippocampal population sparsity (Fig. 5e), we considered the 5-s epochs immediately before and during yellow-laser-on periods in abDGC::GFP and abDGC::ArchT mice to estimate sparsity as described above for optogenetic stimulation, again performing a paired analysis on the average sparsity for each recording session.

**Extended Data Fig. 9:**
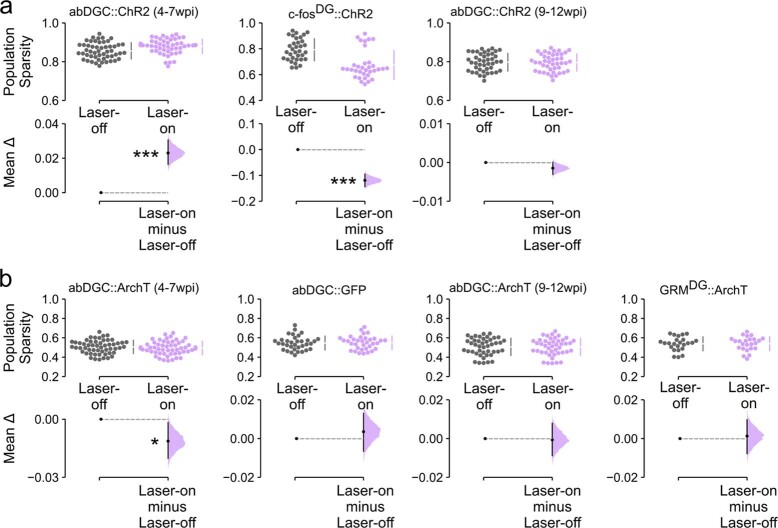
Alternative measure of population sparsity using the Hoyer method. To corroborate the results we obtained using the Gini index, the population-level sparsity was also assessed using the Hoyer method^77,79^. For each estimation plot, each data point represents the mean sparsity of hippocampal PC population vectors for one recording session (same population vectors, same sessions as those used with the Gini index). **(a)** From left to right: population sparsity before (laser-off, gray) versus immediately following (laser-on, purple) optogenetic activation of 4-7-week-old abDGCs (see Fig. 2g), a set of DG cells recruited irrespective of their birthdate (see Fig. 3c), and 9-12-week-old abDGCs (see Fig. 4c) (n = 52 sessions in 8 mice, n = 34 sessions in 3 mice, n = 43 sessions in 3 mice, respectively). **(b)** From left to right: population sparsity before (laser-off, gray) versus following (laser-on, purple) light onset in abDGC::ArchT and abDGC::GFP mice for 4-7-week-old or 9-12-week-old abDGC silencing (see Fig. 5e), and in Grm2^DG^::ArchT mice (see Extended Data Fig. 4i) (n = 54 sessions in 4 mice, n = 34 sessions in 2 mice, n = 44 sessions in 2 mice, n = 24 sessions in 3 mice, respectively). Panels a,b show Cumming estimation plots as described in Extended Data Fig. 1e. ***p < 0.001, *p < 0.05.

To cross-validate the results obtained using the Gini index, we also calculated population-level sparsity using the Hoyer method^77,79^ as:

$$S = \frac{{\sqrt {{{\mathrm{N}}}} - \left( {\mathop {\sum }\nolimits_i |x_i|} \right)/\sqrt {\mathop {\sum}\nolimits_i {x_i^2} } }}{{\sqrt N - 1}}$$

where *x* is the population vector containing the spike counts discharged by each PC in a given time window; *N* is the length of that vector (that is, the number of simultaneously recorded PCs; and *i* refers to each member of *x*. These additional analyses are presented in Extended Data Fig. 9.

### Effects of optogenetic silencing of abDGCs on hippocampal spiking activity

To investigate the effect of silencing abDGCs on the spiking activity of the other hippocampal neurons, we generated PSTHs for each neuron recorded from abDGC::ArchT and abDGC::GFP mice with reference to the onset of light delivery. From the PSTHs, we calculated the *Z*-scored change in spike counts during light delivery, relative to a 5-s baseline before laser-onset, in 875-ms time bins. For analysis, we summed the *Z*-scores during laser-on periods to generate a single value for the change in firing rate for each neuron (Fig. 5d). For display, the group means were smoothed with a three-point moving-average filter (Fig. 5b,c and Extended Data Fig. 4e,h). To calculate the absolute change in firing rate, we summed all spikes in laser-on versus laser-off periods and divided these counts by the respective durations (Extended Data Fig. 4b). To visualize the variability of responses, we also plotted kernel density estimates^80^ for the distribution of summed *Z*-scores for all hippocampal cell types in abDGC::GFP and abDGC::ArchT mice (Extended Data Fig. 4a,d).

### Theta-paced ensemble co-firing

To generate population matrices containing temporally correlated spiking of CA3–CA1 cell-pair ensembles, we first detected theta cycles in each recording session. Theta cycles were then used as time windows to generate population firing vectors using the spike counts nested in each theta cycle for all simultaneously recorded CA3 and CA1 PCs (row × column, cells × theta cycles) in each session, such that each row was a spike train from one cell. For further analysis, each matrix had to contain more than ten simultaneously recorded PCs. We then calculated the Pearson correlation coefficient for each CA3–CA1 cell pair to generate a similarity coefficient matrix for co-firing in pairs of simultaneously recorded CA3–CA1 cells (Fig. 6d and Extended Data Figs. 5d and 6d).

### cNOR task

On each task day, mice first explored a circular familiar open field (~15 min) to provide a baseline of hippocampal network activity. Mice then explored a square-walled open field (Fig. 6a,b; the ‘object arena’) that contained four objects on each subsequent session. On the first session in the object arena (‘sampling’ session, 10 min), mice encountered four novel objects. These first-time-seen objects were each positioned midway along a given wall, ~1 cm from the wall edge. On the next session, one of the four objects was replaced with a different and novel object, then allowing the mouse to explore again (‘test’ session, 10 min). This novel object testing process was repeated across the subsequent 10-min sessions until all the objects initially explored in the sampling session had been replaced with different novel objects. On each test session, we measured the time spent exploring each object, and we calculated the percentage time spent investigating the novel object versus the (mean) percentage time spent investigating the familiar objects (that is, those objects seen in the previous session). The inter-trial interval was <5 min, during which the mouse was placed back in its home cage within the recording room. In control mice, this cNOR task layout is prone to proactive interference across test sessions, as illustrated by poorer behavioral performance in the third and fourth tests compared with the first and second tests. Therefore, to draw conclusions about the role of abDGCs in novelty detection without a possible interaction with proactive interference, in this study we analyzed behavioral performance and associated neuronal activity in the first and second tests for both abDGC::ArchT and abDGC::GFP mice. Accordingly, across alternating task days we applied DG-targeting light delivery on either the first or the second test (Fig. 6b; laser-on tests: 30-s light pulses, 561 nm, with random inter-pulse intervals of 5–22 s). This way, in each group of mice, we obtained equal numbers of first and second test sessions with laser-on and laser-off. The cNOR procedure for the hippocampal-lesioned and sham-lesioned mice was similar, with no DG light delivery.

### Population dimensionality

We estimated the dimensionality of the PC population firing structure using the theta-paced activity matrices generated as described above (Theta-paced ensemble co-firing). For each laser-off and laser-on session, we then applied principal component analysis to each corresponding mean-centered activity matrix, using the number of simultaneously recorded PCs as the maximum number of components. Each matrix required more than ten PCs for inclusion in the analysis. We then extracted the number of components explaining 80% of the variance in these theta-paced population vectors of instantaneous PC firing (Fig. 6e). We used all PCs recorded on each recording day in this analysis. That is, the lengths of the population vectors forming the activity matrices compared between laser-off and laser-on sessions of that day are identical (that is, they contained the same PCs). For each day, we then calculated the number of principal components in laser-on and laser-off tests and divided these by the number of neurons in each vector (dimensionality score = estimated dimensionality/number of PCs in each vector). Additional analyses of dimensionality for various levels of explained variance (70%, 75%, 85% and 90%) can be found in Extended Data Fig. 8. Note that 'PC' refers to principal cells and not principal components throughout this study (e.g., in Extended Data Fig. 8).

### Tissue processing and immunohistochemistry

At the completion of experiments, mice were deeply anesthetized with pentobarbital and perfused transcardially with 0.1 M PBS followed by 4% paraformaldehyde (PFA) in PBS. Brains were extracted and kept in 4% PFA for ~24–72 h and then transferred to PBS (with 0.05% sodium-azide). For immunostaining, free-floating sections (50-µm) were rinsed in PBS with 0.25% Triton X-100 (PBS-T) and were blocked for 1 hour at ~20 °C in PBS-T with 10% normal donkey serum (NDS). Sections were then incubated with primary antibodies diluted in 3% NDS blocking solution and incubated at 4 °C for 72 hours (Prox1 anti-rabbit, 1:1,000, AngioBio, catalog no. 11-00P; GFP anti-chicken, 1:1,000, Aves Labs, catalog no. GFP-1020; NeuN guinea pig, 1:500, Synaptic Systems, catalog no. 266 004). All sections were rinsed three times for 15 min in PBS-T and incubated for 4 hours at ~20 °C in secondary antibodies in the blocking solution (Cy3 donkey anti-rabbit, 1:1,000, Jackson ImmunoResearch, catalog no. 711-165-152; Cy3 donkey anti-guinea pig, 1:400, Jackson ImmunoResearch, catalog no. 706-165-148; goat anti-chicken 488, 1:1,000, Thermo Fisher Scientific, catalog no. A-11039). Sections were then rinsed three times for 15 min in PBS-T, with some sections then incubated for 1 min with DAPI (0.5 µg ml^−1^, Sigma, D8417) diluted in PBS to label cell nuclei before three additional rinse steps of 10 min each in PBS. Sections were mounted on slides, cover-slipped with Vectashield (Vector Laboratories, catalog no. H-1000) and stored at 4 °C. Sections were also used for anatomical verification of the tetrode tracks. Images were acquired using a Zeiss confocal microscope (LSM 880 Indimo, Axio Imager 2) with a Plan-Apochromat ×20/0.8 M27 objective and the ZEN (Zeiss Black 2.3) software. For the hippocampal lesion experiment, mice were perfused transcardially with physiological saline (0.9% NaCl) followed by 10% formol saline (10% formalin in physiological saline). The brains were then removed and placed in 10% formol saline and 72 hours later transferred to 30% sucrose-formalin. Coronal sections (50 µm) were cut on a freezing microtome and stained with cresyl violet to enable visualization of lesion extent.

### Stereological analysis

The number of cells expressing the Cre-dependent ChR2-eYFP construct in the dorsal DG of both abDGC::ChR2 mice and c-fos^DG^::ChR2 mice was estimated using the optical fractionator method on a systematic random sampling of every fifth section along the rostro-caudal axis of the hippocampal formation^81^. On each section, ChR2-eYFP cells were counted in the granular and subgranular layers, excluding those in the outermost focal plane. Resulting numbers were tallied and multiplied by the inverse of the section-sampling fraction (1/ssf = 5).

### Statistical analysis

Analyses were performed in Python v.3.6 (https://www.python.org/downloads/release/python-363/), using the Python packages DABEST^82^, scipy^83^, numpy^84^, matplotlib^85^, seaborn^86^, pandas^87^ and scikit-learn^88^. Error bars, mean ± s.e.m. unless otherwise stated. We used throughout this study a bootstrap-coupled estimation of effect sizes, plotting the data against a mean (median or paired mean, as indicated) difference between the left-most condition and one or more conditions on the right (right *y* axis), and compared this difference against zero using 5,000 bootstrapped resamples. In these estimation graphics (DABEST plots^82^), each black dot indicates a mean (median or mean paired, as indicated in the right *y*-axis label) difference and the associated black ticks depict error bars representing 95% confidence intervals; the shaded area represents the bootstrapped sampling-error distribution. Bandwidth estimates for the kernel density estimate were computed using the scikit-learn package. All statistical tests were performed two-sided, unless otherwise stated, using the estimation statistics framework. Paired permutation tests (or equivalent paired tests) were performed for repeated-measures analyses (for example, comparing responses in the same neurons at two different time points) and unpaired tests used for independent samples (for example, comparing responses across different populations of neurons). No statistical methods were used to pre-determine sample sizes, but our sample sizes are similar to those reported in previous publications (for example, see refs. ^12–14,16^). Data distribution was assumed to be normal, but this was not formally tested. Mice were randomly allocated to ArchT and GFP-only groups. In the novel object recognition task, objects and their positions and the order of their replacement were randomized. Data collection could not be performed blind to the conditions of the experiments since the experimenters had to be aware as to which conditions they had to expose each mouse on a given day and on a given session (for example, Light delivery OFF versus ON). Neural and behavioral data analyses were conducted in an identical way regardless of the identity of the experimental condition from which the data were collected, with the investigators blind to group allocation during data analysis of experiments (for example, Light delivery OFF versus ON). No mice were excluded. Inclusion criteria for well-isolated single units were used as published in previous studies and described in the methods section. For population dimensionality analysis, the recording day had to contain >10 simultaneously recorded PCs for inclusion.

### Reporting summary

Further information on research design is available in the Nature Research Reporting Summary linked to this article.

## Online content

Any methods, additional references, Nature Research reporting summaries, source data, extended data, supplementary information, acknowledgements, peer review information; details of author contributions and competing interests; and statements of data and code availability are available at 10.1038/s41593-022-01176-5.

## Supplementary information

Supplementary Information Supplementary Tables 1 and 2.

Reporting Summary

## Data Availability

The datasets generated during and/or analyzed during the current study will be made available via the MRC BNDU Data Sharing Platform (https://data.mrc.ox.ac.uk/) on reasonable request.

## Code availability

The software used for data acquisition and analysis is available using the web links mentioned in the Methods.
