# Supplementary material for: Adult-born dentate granule cells promote hippocampal population sparsity
Source: Nat Neurosci. 2022 Oct 10;25(11):1481–91. doi: 10.1038/s41593-022-01176-5 (PMC9630129; doi:10.1038/s41593-022-01176-5)
Supplement: Supplementary file 1 — Supplementary Tables 1 and 2. [file 41593_2022_1176_MOESM1_ESM.pdf]

nature neuroscience

Article

https://doi.org/10.1038/s41593-022-01176-5

Adult-born dentate granule cells promote hippocampal population sparsity

In the format provided by the authors and unedited

Adult-born dentate granule cells promote hippocampal population sparsity

Stephen B. McHugh, Vítor Lopes-dos-Santos, Giuseppe P. Gava, Katja Hartwich, Shu Kit Eric Tam, David M. Bannerman, David Dupret

Supplementary Information

Supplementary Table 1

Group	Strain / genotype	Viruses injected	Testing/recording window (wpi)	# Mice
abDGC::ChR2	Nestin-cre B6.Cg-Tg(Nes-cre)1Kln/J	AAV-EF1a-DIO-hChR2(E123T/T159C)-EYFP	4-7	6
abDGC::ChR2	C57BL6/J	(1) MMLV-pMX-T2A-Cre-mCherry<br>(2) AAV-EF1a-DIO-hChR2(E123T/T159C)-EYFP	4-7	2
c-fos^{DG}::ChR2	c-fos tTA	(1) pAAV-TRE3G-FLAG-Cre<br>(2) AAV-EF1a-DIO-hChR2(E123T/T159C)-EYFP	n/a	3
abDGC::ChR2	C57BL6/J	(1) MMLV-pMX-T2A-Cre-mCherry<br>(2) AAV-EF1a-DIO-hChR2(E123T/T159C)-EYFP	9-12	3
abDGC::ArchT	C57BL6/J	(1) MMLV-pMX-T2A-Cre-mCherry<br>(2) AAV-CAG-Flex-ArchT-GFP (UNC AV6222b)	4-7	4
abDGC::ArchT	C57BL6/J	(1) MMLV-pMX-T2A-Cre-mCherry<br>(2) AAV-CAG-Flex-ArchT-GFP (UNC AV6222b)	9-12	2
abDGC::GFP	C57BL6/J	(1) MMLV-pMX-T2A-Cre-mCherry<br>(2) AAV-CAG-Flex-GFP (UNC AV4530B)	n/a	2
Grm2^{DG}::ArchT	Tg(Grm2-cre) MR90Gsat/Mmucd	AAV-CAG-Flex-ArchT-GFP (UNC AV6222b)	n/a	4

Supplementary Table 2

Figure	# sessions	# mice	# Principal Cell used in analyses			
			**abDGC**	**DG**	**CA3**	**CA1**
Fig. 1f,h	62	8	33 (recorded in 16/62 sessions)<br>Mean = 4.1 abDGCs per mouse	887	n/a	n/a
Fig. 2d	16	8	33	276	43	266
Fig. 2g	52	8	n/a	197	21	263
Fig. 3c	34	3	n/a	7	12	385
Fig. 4c	43	3	n/a	160	102	463
Fig. 4d	62 (abDGC::ArchT 4-7wpi)<br>43 (abDGC::ArchT 9-12 wpi)	8<br>3	n/a<br>n/a	883<br>160	195<br>102	741<br>463
Fig. 5d,e	34 (abDGC::GFP)<br>54 (abDGC::ArchT4-7wpi)<br>44 (abDGC::ArchT 9-12wpi)	2<br>4<br>2	n/a<br>n/a<br>n/a	147<br>219<br>131	70<br>290<br>56	124<br>437<br>213
