# Supplementary material for: Adult-born dentate granule cells promote hippocampal population sparsity
Source: Nat Neurosci. 2022 Oct 10;25(11):1481–91. doi: 10.1038/s41593-022-01176-5 (PMC9630129; doi:10.1038/s41593-022-01176-5)
Supplement: Supplementary file 2 — Reporting Summary [file 41593_2022_1176_MOESM2_ESM.pdf]

Corresponding author(s): Stephen B. McHugh and David Dupret

Last updated by author(s): Aug 26, 2022

Reporting Summary

Nature Research wishes to improve the reproducibility of the work that we publish. This form provides structure for consistency and transparency in reporting. For further information on Nature Research policies, see our Editorial Policies and the Editorial Policy Checklist.

Statistics

For all statistical analyses, confirm that the following items are present in the figure legend, table legend, main text, or Methods section.

n/a	Confirmed	
☐	☒	The exact sample size (n) for each experimental group/condition, given as a discrete number and unit of measurement
☐	☒	A statement on whether measurements were taken from distinct samples or whether the same sample was measured repeatedly
☐	☒	The statistical test(s) used AND whether they are one- or two-sided<br>*Only common tests should be described solely by name; describe more complex techniques in the Methods section.*
☐	☒	A description of all covariates tested
☐	☒	A description of any assumptions or corrections, such as tests of normality and adjustment for multiple comparisons
☐	☒	A full description of the statistical parameters including central tendency (e.g. means) or other basic estimates (e.g. regression coefficient) AND variation (e.g. standard deviation) or associated estimates of uncertainty (e.g. confidence intervals)
☐	☒	For null hypothesis testing, the test statistic (e.g. F, t, r) with confidence intervals, effect sizes, degrees of freedom and P value noted<br>*Give P values as exact values whenever suitable.*
☒	☐	For Bayesian analysis, information on the choice of priors and Markov chain Monte Carlo settings
☒	☐	For hierarchical and complex designs, identification of the appropriate level for tests and full reporting of outcomes
☐	☒	Estimates of effect sizes (e.g. Cohen's d, Pearson's r), indicating how they were calculated

Our web collection on statistics for biologists contains articles on many of the points above.

Software and code

Policy information about availability of computer code

Data collection	Neural data was acquired using the integrated circuit RHD2164 from Intan Technologies; and unit isolation was performed using Kilosort 1.0 via the Spikeforest (v1) sorting framework. Confocal images were acquired using the ZEN (Zeiss Black 2.3) software.
Data analysis	Data were analyzed in Python 3.6 and and using the packages DABEST, scikit-learn 0.22.1, Numpy 1.18.2, Scipy 1.4.1, Matplotlib 3.2.1, Pandas 1.1.5, Seaborn 0.11.1 and Empirical Mode Decomposition emd 0.5.5.

For manuscripts utilizing custom algorithms or software that are central to the research but not yet described in published literature, software must be made available to editors and reviewers. We strongly encourage code deposition in a community repository (e.g. GitHub). See the Nature Research guidelines for submitting code & software for further information.

Data

Policy information about availability of data

All manuscripts must include a data availability statement. This statement should provide the following information, where applicable:

- Accession codes, unique identifiers, or web links for publicly available datasets
- A list of figures that have associated raw data
- A description of any restrictions on data availability

The datasets generated during and/or analysed during the current study will be made available via the MRC BNDU Data Sharing Platform (https://data.mrc.ox.ac.uk/) on reasonable request.

Field-specific reporting

Please select the one below that is the best fit for your research. If you are not sure, read the appropriate sections before making your selection.

☒ Life sciences ☐ Behavioural & social sciences ☐ Ecological, evolutionary & environmental sciences

For a reference copy of the document with all sections, see nature.com/documents/nr-reporting-summary-flat.pdf

Life sciences study design

All studies must disclose on these points even when the disclosure is negative.

Sample size	The dataset includes n=5,158 principal cells and n=361 interneurons recorded from the hippocampus. A total of 26 mice were used in the electrophysiology experiments, and a further 16 in the lesion experiment. No statistical methods were used to pre-determine sample sizes but our sample sizes are similar to those reported in previous publications (e.g. ref 12, 13, 14, 16).
Data exclusions	No mice were excluded. Inclusion criteria for well-isolated single units were used as published in previous studies and described in the methods section. For population dimensionality analysis, the recording day had to contain >10 simultaneously recorded principal cells for inclusion.
Replication	The influence of adult-born dentate granule cells (abDGCs) on population sparsity was replicated across four independent mouse cohorts (abDGC-chr2 mice and abDGC-archT mice), when abDGCs increased population sparsity selectively when activated in their 4-7-week, but not 9-12-week, post-birth period; likewise, silencing 4-7-week-old; but not 9-12-week-old, abDGCs decreased population sparsity.
Randomization	Mice were randomly allocated to ArchT and GFP-only groups. In the novel object recognition task, objects and their positions and the order of their replacement was randomized.
Blinding	Data collection could not be performed blind to the conditions of the experiments since the experimenters had to be aware as to which conditions they had to expose each mouse on a given day and on a given session (e.g. Light-delivery OFF versus ON). Neural and behavioural data analyses were conducted in an identical way regardless of the identity of the experimental condition from which the data were collected, with the investigators blind to group allocation during data analysis of experiments (e.g. Light-delivery OFF versus ON).

Reporting for specific materials, systems and methods

We require information from authors about some types of materials, experimental systems and methods used in many studies. Here, indicate whether each material, system or method listed is relevant to your study. If you are not sure if a list item applies to your research, read the appropriate section before selecting a response.

Materials & experimental systems

n/a	Involved in the study	
☐	☒	Antibodies
☒	☐	Eukaryotic cell lines
☒	☐	Palaeontology and archaeology
☐	☒	Animals and other organisms
☒	☐	Human research participants
☒	☐	Clinical data
☒	☐	Dual use research of concern

Methods

n/a	Involved in the study	
☒	☐	ChIP-seq
☒	☐	Flow cytometry
☒	☐	MRI-based neuroimaging

Antibodies

Antibodies used	The following antibodies were used for immunofluorescence staining: Prox1 anti-rabbit, AngioBio, Catalog# 11-00P; GFP anti-chicken, Aves Labs, Catalog# GFP-1020; NeuN guinea pig, Synaptic Systems Catalog# 266 004; Cy3 donkey anti-rabbit, Jackson ImmunoResearch Catalog# 711-165-152; Cy3 donkey anti-guinea pig, Jackson ImmunoResearch Catalog# 706-165-148; goat anti-chicken 488, Thermo Fisher Scientific, Catalog# A-11039.
Validation	These antibodies have been validated for immunostaining by the company and/or studies cited on company's website:<br>anti-Prox1 (AngioBio, #11-00P): http://www.angiobio.com/new/product.php?pid=2<br>anti-GFP (Aves Labs, #GFP-1020): https://www.aveslabs.com/products/anti-green-fluorescent-protein-antibody-gfp<br>anti-NeuN (Synaptic Systems, #266 004): https://sysy.com/product/266004<br>Cy3 donkey anti-rabbit (Jackson ImmunoResearch, #711-165-152): https://www.jacksonimmuno.com/catalog/products/711-165-152<br>Cy3 donkey anti-guinea pig (Jackson ImmunoResearch, #706-165-148): https://www.jacksonimmuno.com/catalog/products/706-165-148<br>Alexa 488 goat anti-chicken (Thermo Fisher Scientific, #A-11039): https://www.thermofisher.com/antibody/product/Goat-anti-Chicken-IgY-H-L-Secondary-Antibody-Polyclonal/A-11039

Animals and other organisms

Policy information about studies involving animals; ARRIVE guidelines recommended for reporting animal research

Laboratory animals	These experiments used adult male C57BL/6J mice (Charles River Laboratories, UK) or transgenic Nestin-cre mice (The Jackson Laboratories; B6.Cg-Tg(Nes-cre)Kln/J, stock number 003771, RRID: IMSR_JAX:003771; maintained on a C57BL/6J background), Grm2-Cre (MMRRC; Tg(Grm2-cre)MR90Gsat/Mmucd; stock # 034611-UCD, RRID:MMRRC_034611-UCD; maintained on a C57BL/6J background) or cFos-tTA (The Scripps Research Institute and maintained at Tufts University). Mice were housed with their littermates until the surgical procedure with free access to food and water in a room with a 12/12h light/dark cycle, 19–23°C ambient temperature and 40–70% humidity. Mice were 4-10 months old at the time of testing.
Wild animals	No wild animals were used in the study.
Field-collected samples	No field collected samples were used in the study.
Ethics oversight	Experimental procedures performed on mice in accordance with the Animals (Scientific Procedures) Act, 1986 (United Kingdom), with final ethical review by the Animals in Science Regulation Unit of the UK Home Office.

Note that full information on the approval of the study protocol must also be provided in the manuscript.
